# Supplementary material for: Population Health at the Academic Health Center: An Interactive, Multipart, Case-Based Session for Executives, Faculty, and Administrators
Source: MedEdPORTAL. 2022 Jan 7;18:11204. doi: 10.15766/mep_2374-8265.11204 (PMC8738160; doi:10.15766/mep_2374-8265.11204)
Supplement: Supplementary file 1 — Call for Abstracts.docxReviewer Rubric.docxCase Stem and Small-Group Prompts.docxSession Evaluation.docxIntroduction to Population Health.pptxFacilitator Guide.docx [file mep_2374-8265.11204-s001.zip › A. Call for Abstracts.docx]

Call for Abstracts

A call for abstracts for the Population Health at the Academic Health Center: An interactive multi-part, case-based session for executives, faculty and administrators.

Dear Colleagues,

Academic Health Center / Association/Institution X is pleased to announce a call for abstractss to share current practices in population health at academic health centers. Population health has been defined as a focus on the health of all people in a given geographic area that emphasizes multi-sector approaches and, in addition to traditional medical care, the incorporation of nonclinical interventions to address social determinants of health.

The presentation format will be brief "ignite talks" limited to five minutes and no more than five slides total. In the spirit of learning from each other, presentations should report not only initiative successes, but elements that surprised and challenged your academic health center and how they were addressed. The goal of this session is to introduce participants to a variety of population health initiatives, with the opportunity to learn more through direct communication with presenters.

The deadline for proposals is *insert date*. Presentations will be made virtually on *insert date* at a time to be determined. Submit your abstracts today!
