# Supplementary material for: Population Health at the Academic Health Center: An Interactive, Multipart, Case-Based Session for Executives, Faculty, and Administrators
Source: MedEdPORTAL. 2022 Jan 7;18:11204. doi: 10.15766/mep_2374-8265.11204 (PMC8738160; doi:10.15766/mep_2374-8265.11204)
Supplement: Supplementary file 1 — Call for Abstracts.docxReviewer Rubric.docxCase Stem and Small-Group Prompts.docxSession Evaluation.docxIntroduction to Population Health.pptxFacilitator Guide.docx [file mep_2374-8265.11204-s001.zip › B. Reviewer Rubric.docx]

Reviewer Rubric

Rubric utilized to score abstracts received for the Population Health at the Academic Health Center: An interactive multi-part, case-based session for executives, faculty and administrators

Reviewer Name:

Abstract Number:

The problem addressed by the initiative is of interest to attendees.

Agree

Somewhat Agree

Somewhat Disagree

Disagree

The description of how the initiative was implement is clear.

Agree

Somewhat Agree

Somewhat Disagree

Disagree

The achieved or intended outcomes are relevant to improving population health at an academic health center

Agree

Somewhat Agree

Somewhat Disagree

Disagree

#### The lessons learned and how they were addressed, will help attendees move forward their thinking about population health

Agree

Somewhat Agree

Somewhat Disagree

Disagree

#### Should this be a podium presentation, poster presentation, or neither?

Podium Presentation

Poster Presentation

Neither

Comments (optional)
