# Supplementary material for: Population Health at the Academic Health Center: An Interactive, Multipart, Case-Based Session for Executives, Faculty, and Administrators
Source: MedEdPORTAL. 2022 Jan 7;18:11204. doi: 10.15766/mep_2374-8265.11204 (PMC8738160; doi:10.15766/mep_2374-8265.11204)
Supplement: Supplementary file 1 — Call for Abstracts.docxReviewer Rubric.docxCase Stem and Small-Group Prompts.docxSession Evaluation.docxIntroduction to Population Health.pptxFacilitator Guide.docx [file mep_2374-8265.11204-s001.zip › D. Session Evaluation.docx]

Session Evaluation

The session evaluation for attendees of the Population Health at the Academic Health Center: An interactive multi-part, case-based session for executives, faculty and administrators. If desired, this evaluation could be tailored for use at a single academic health center by inserting the name of the institution in place of the generic “academic health center” and making small grammatical edits.

Thank you for attending the session on Population Health Initiatives held on *insert date*.  We appreciate your participation and hope you found the session valuable. 

We continually aim to improve programming. To inform our future efforts, please complete the below 10-question survey. We estimate that it will take less than 5 minutes. Thank you for your time and consideration.

Top of Form

What is your role at your academic health center?

Bottom of Form

#### This population health session was valuable to my role at my academic health center.

Strongly Agree

Agree

Neutral

Disagree

Strongly Disagree

#### I will apply knowledge gained from this population health session at my academic health center.

Strongly Agree

Agree

Neutral

Disagree

Strongly Disagree

#### Hearing colleagues describe population health initiatives at their academic health center provided me ideas to implement at my own academic health center.

####

Strongly Agree

Agree

Neutral

Disagree

Strongly Disagree

#### I will follow-up with members of my academic health center regarding information shared in this population health session.

Strongly Agree

Agree

Neutral

Disagree

Strongly Disagree

#### Which population health breakout session did you attend?

Analytics

Community Engagement

Education and Training

Implementation

#### Before the population health breakout session, I appreciated how the structure of an academic health center could impact its role in promoting population health

Strongly Agree

Agree

Neutral

Disagree

Strongly Disagree

#### After the population health breakout session, I appreciated how the structure of an academic health center could impact its role in promoting population health.

Strongly Agree

Agree

Neutral

Disagree

Strongly Disagree

#### The breakout session provided me ideas for how to leverage the structure of my academic health center to impact population health

Strongly Agree

Agree

Neutral

Disagree

Strongly Disagree

#### Please comment on which aspect of the population health session you found most and least effective.
