# Supplementary material for: Population Health at the Academic Health Center: An Interactive, Multipart, Case-Based Session for Executives, Faculty, and Administrators
Source: MedEdPORTAL. 2022 Jan 7;18:11204. doi: 10.15766/mep_2374-8265.11204 (PMC8738160; doi:10.15766/mep_2374-8265.11204)
Supplement: Supplementary file 1 — Call for Abstracts.docxReviewer Rubric.docxCase Stem and Small-Group Prompts.docxSession Evaluation.docxIntroduction to Population Health.pptxFacilitator Guide.docx [file mep_2374-8265.11204-s001.zip › E. Introduction to Population Health.pptx]

## Slide 1
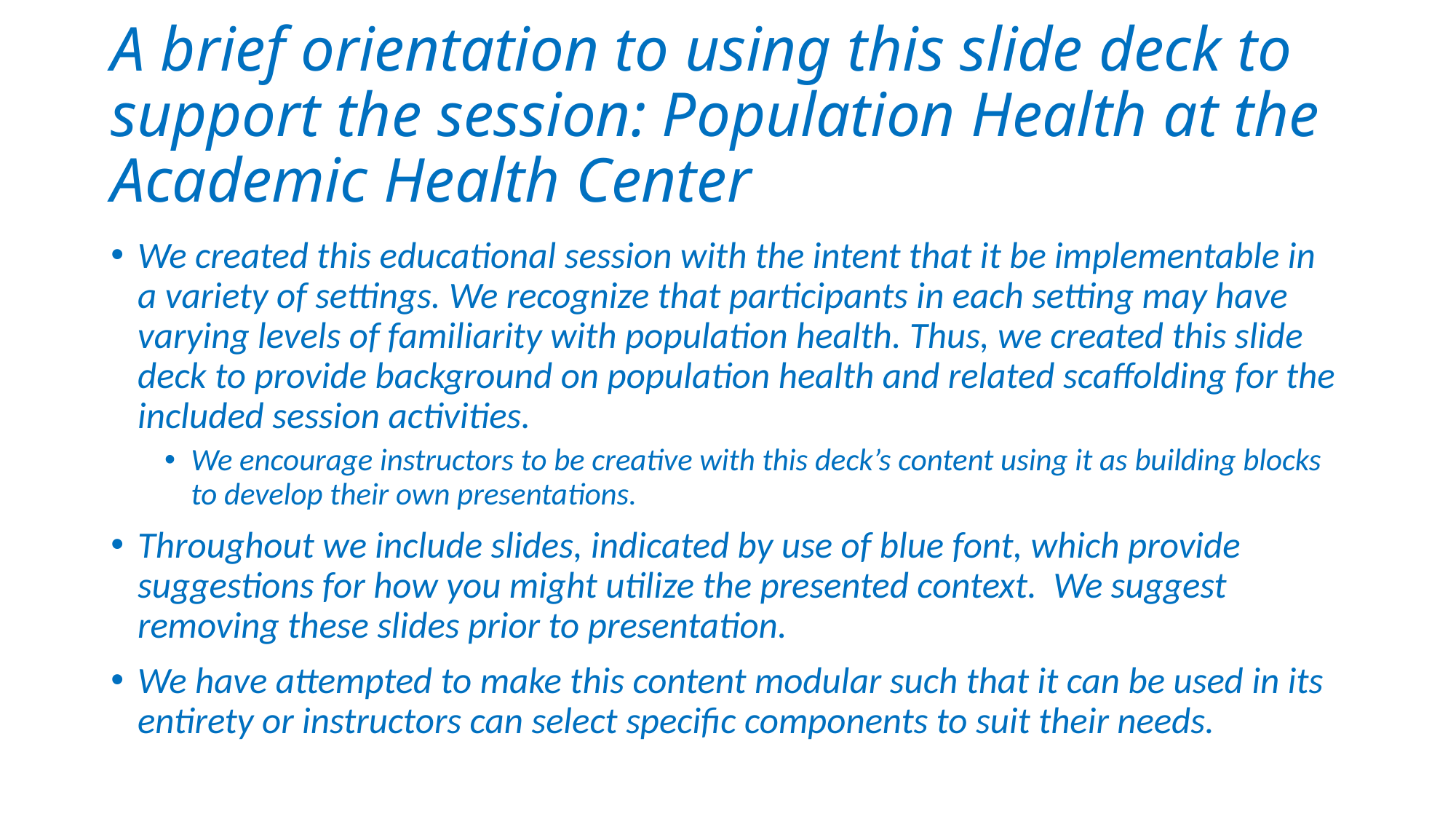

# A brief orientation to using this slide deck to support the session: Population Health at the Academic Health Center
We created this educational session with the intent that it be implementable in a variety of settings. We recognize that participants in each setting may have varying levels of familiarity with population health. Thus, we created this slide deck to provide background on population health and related scaffolding for the included session activities.
We encourage instructors to be creative with this deck’s content using it as building blocks to develop their own presentations.
Throughout we include slides, indicated by use of blue font, which provide suggestions for how you might utilize the presented context. We suggest removing these slides prior to presentation.
We have attempted to make this content modular such that it can be used in its entirety or instructors can select specific components to suit their needs.

## Slide 2
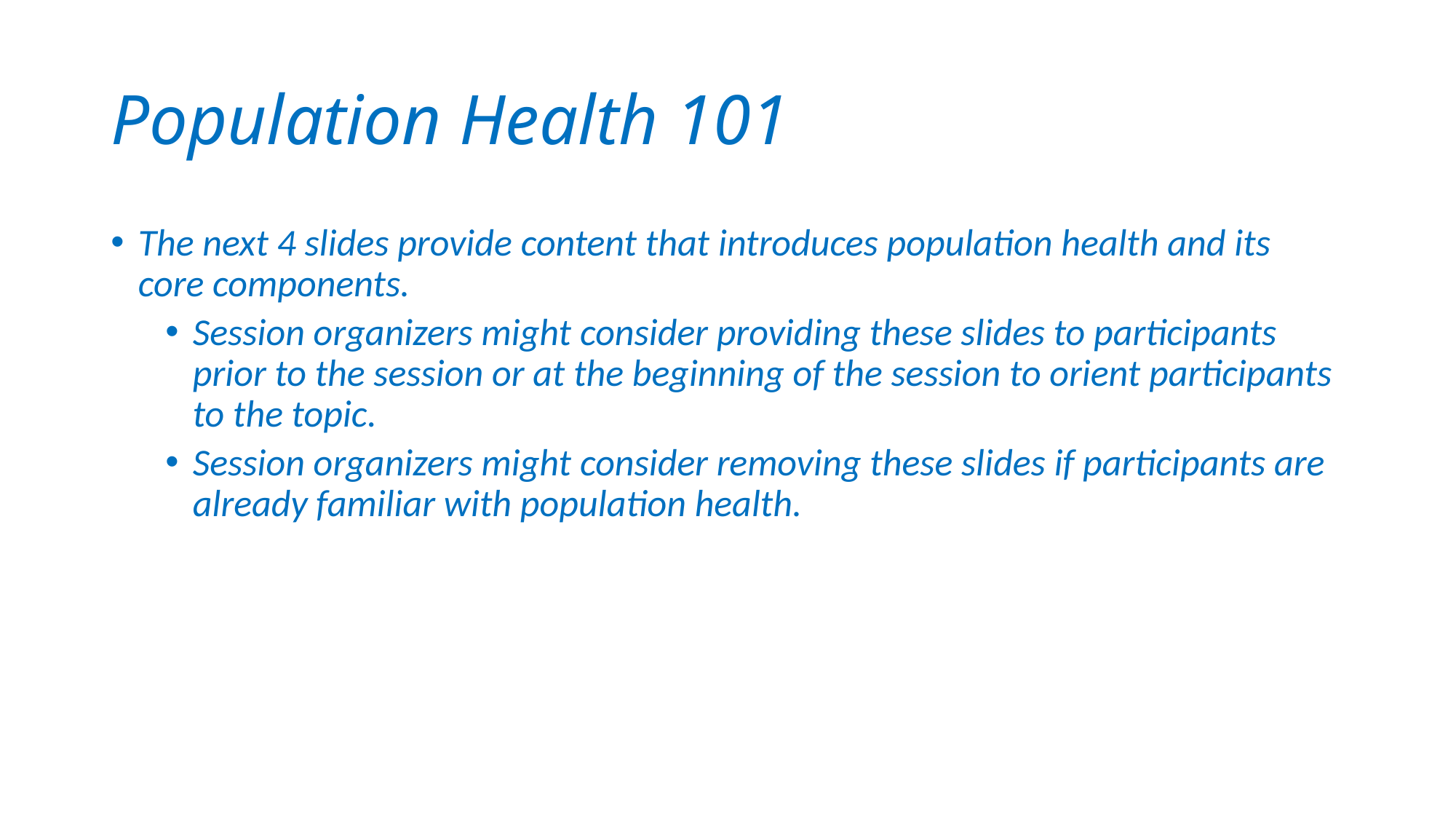

# Population Health 101
The next 4 slides provide content that introduces population health and its core components.
Session organizers might consider providing these slides to participants prior to the session or at the beginning of the session to orient participants to the topic.
Session organizers might consider removing these slides if participants are already familiar with population health.

## Slide 3
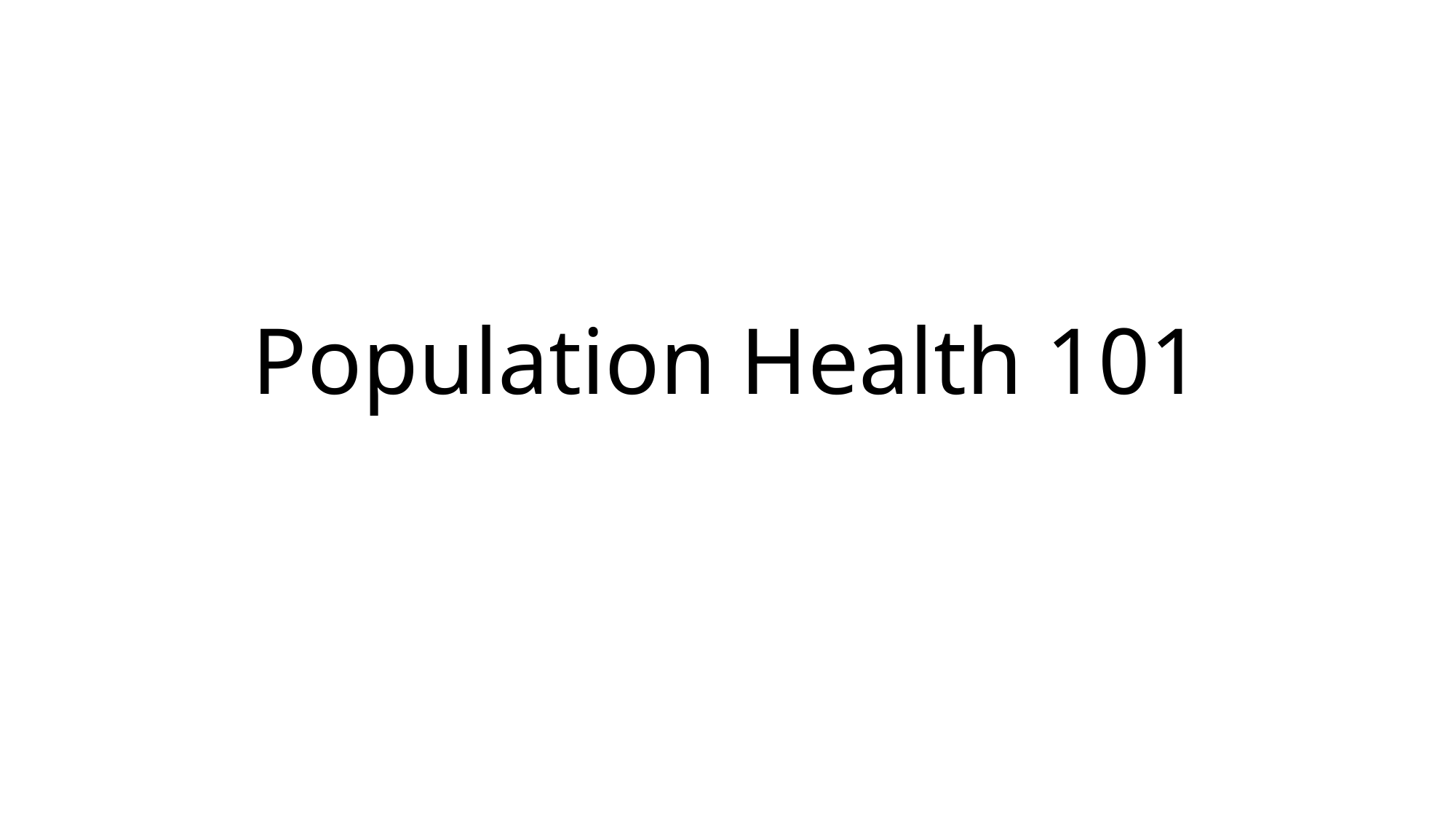

# Population Health 101

## Slide 4
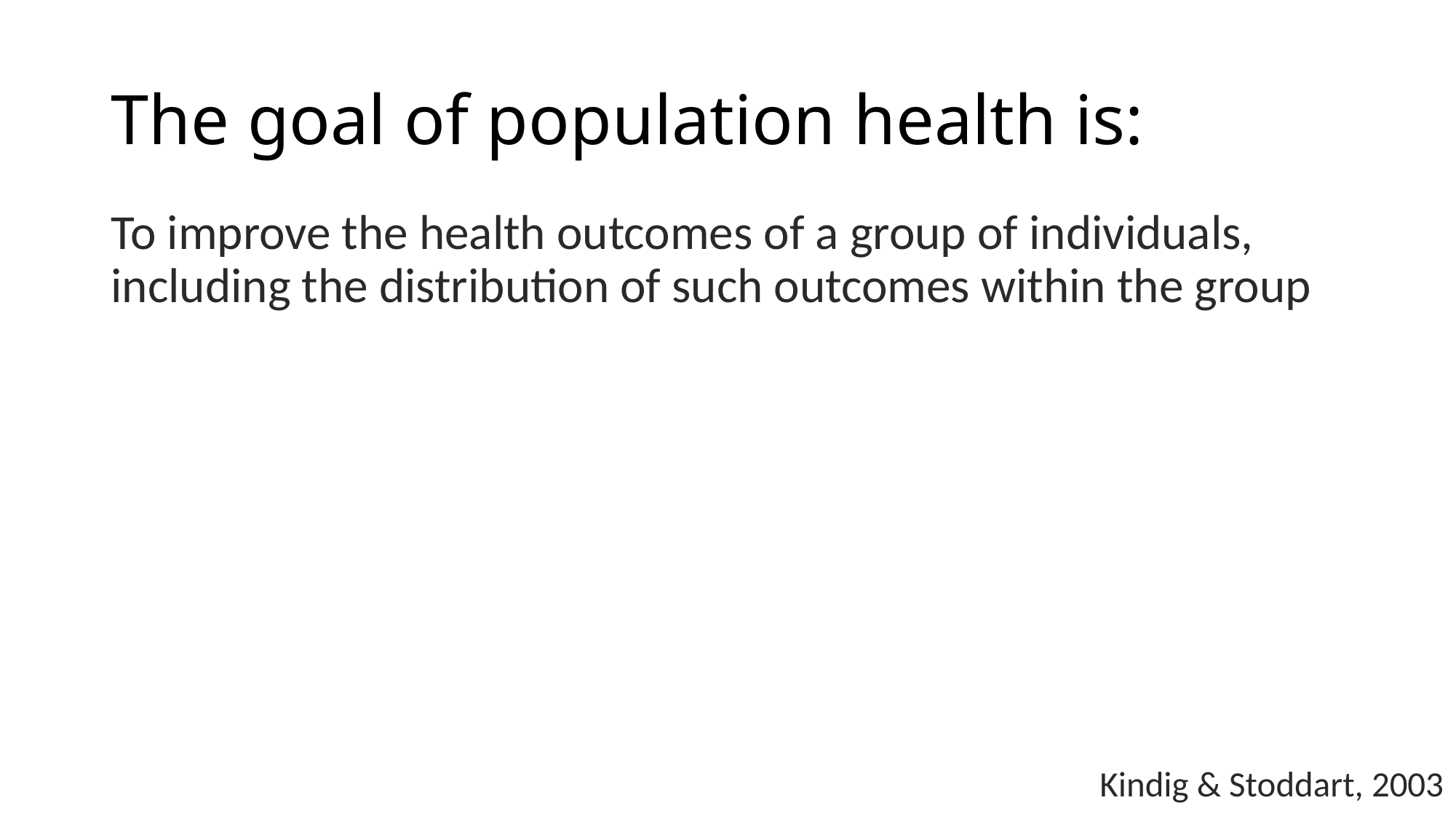

# The goal of population health is:
To improve the health outcomes of a group of individuals, including the distribution of such outcomes within the group
Kindig & Stoddart, 2003

## Slide 5
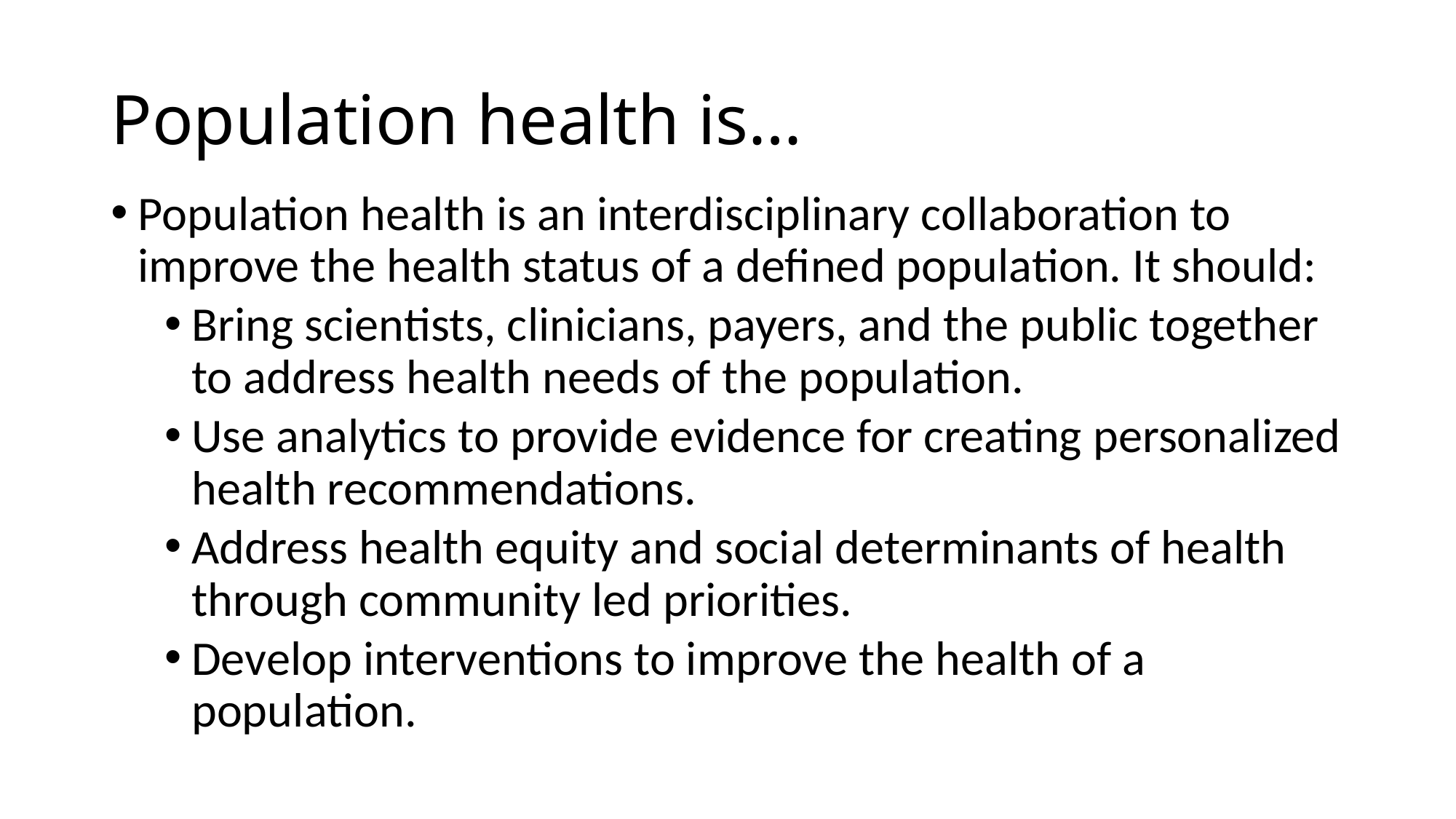

# Population health is…
Population health is an interdisciplinary collaboration to improve the health status of a defined population. It should:
Bring scientists, clinicians, payers, and the public together to address health needs of the population.
Use analytics to provide evidence for creating personalized health recommendations.
Address health equity and social determinants of health through community led priorities.
Develop interventions to improve the health of a population.

## Slide 6
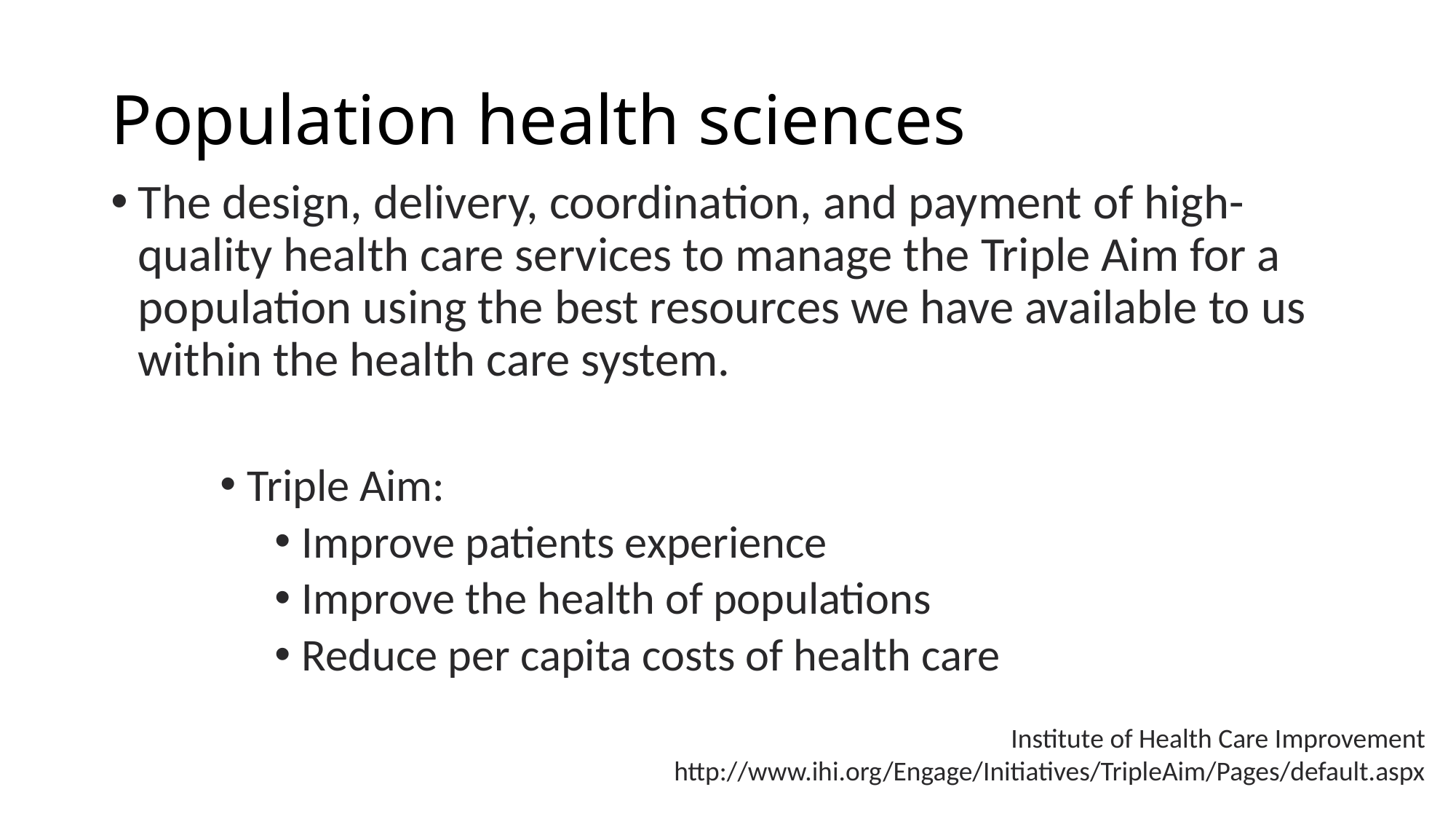

# Population health sciences
The design, delivery, coordination, and payment of high-quality health care services to manage the Triple Aim for a population using the best resources we have available to us within the health care system.
Triple Aim:
Improve patients experience
Improve the health of populations
Reduce per capita costs of health care
Institute of Health Care Improvement
http://www.ihi.org/Engage/Initiatives/TripleAim/Pages/default.aspx

## Slide 7
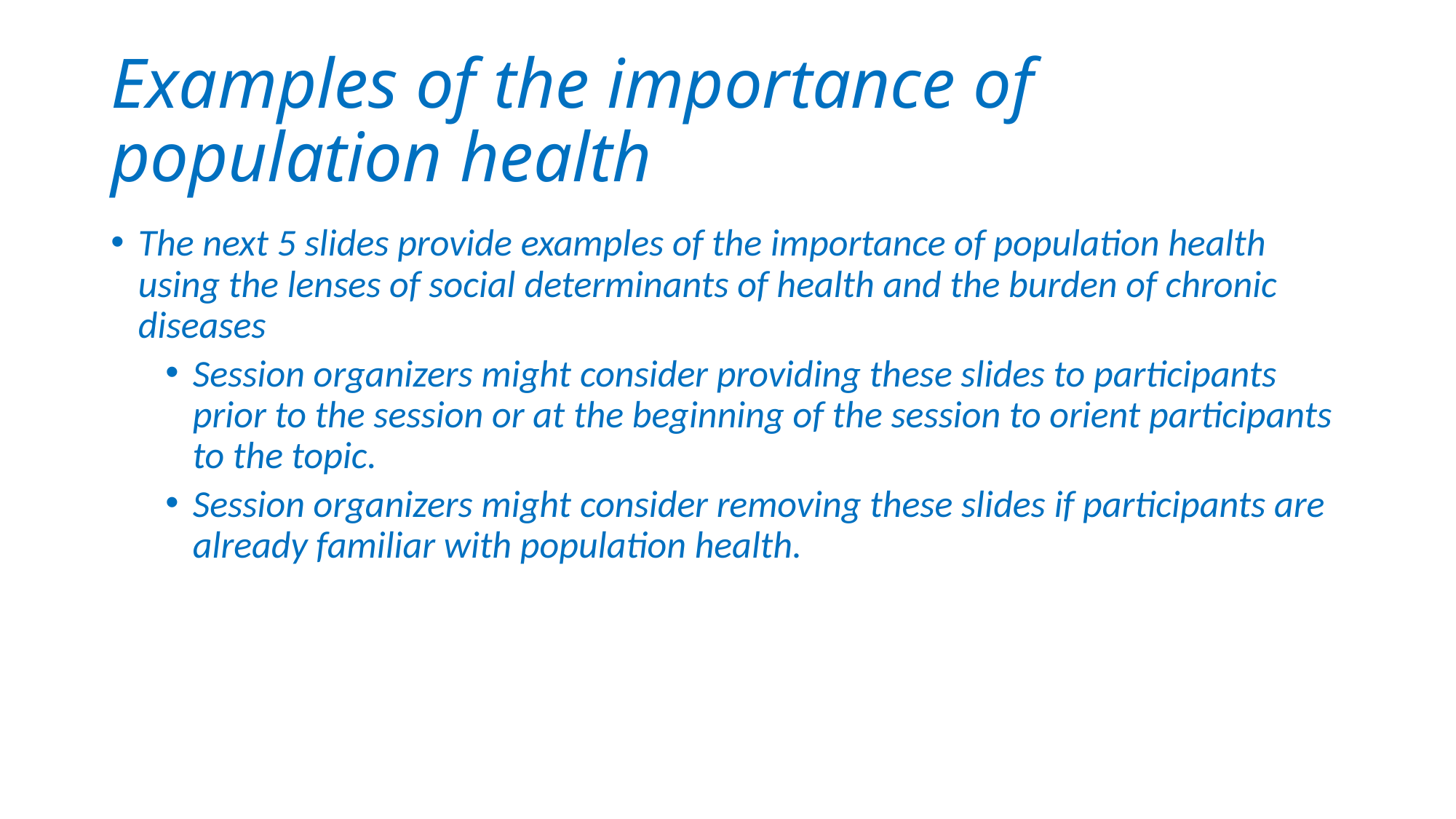

# Examples of the importance of population health
The next 5 slides provide examples of the importance of population health using the lenses of social determinants of health and the burden of chronic diseases
Session organizers might consider providing these slides to participants prior to the session or at the beginning of the session to orient participants to the topic.
Session organizers might consider removing these slides if participants are already familiar with population health.

## Slide 8
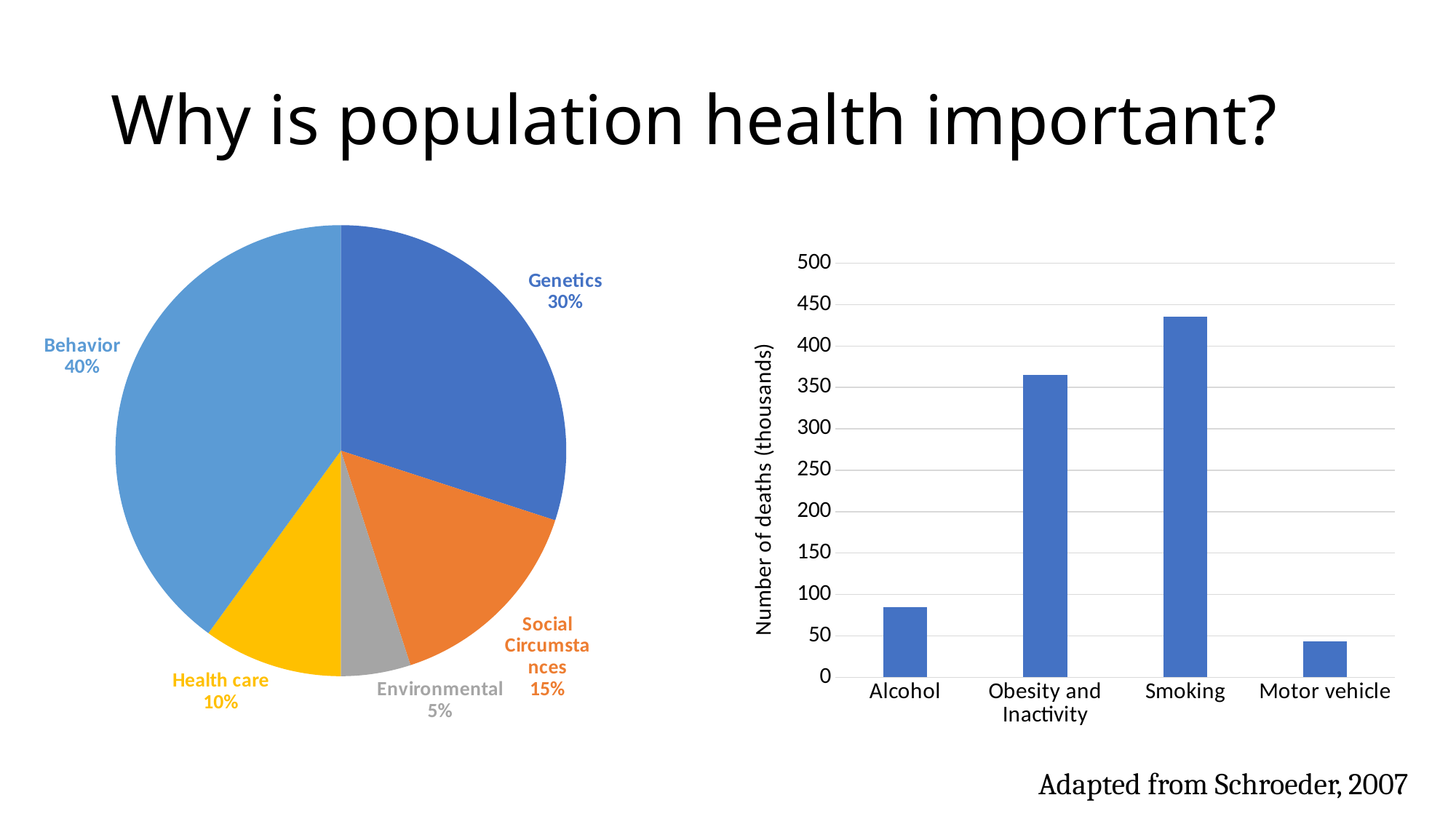

# Why is population health important?
### Chart
| Category | Column1 |
|---|---|
| Genetics | 0.3 |
| Social Circumstances | 0.15 |
| Environmental | 0.05 |
| Health care | 0.1 |
| Behavior | 0.4 |
### Chart
| Category | Series 1 |
|---|---|
| Alcohol | 85.0 |
| Obesity and Inactivity | 365.0 |
| Smoking | 435.0 |
| Motor vehicle | 43.0 |Adapted from Schroeder, 2007

## Slide 9
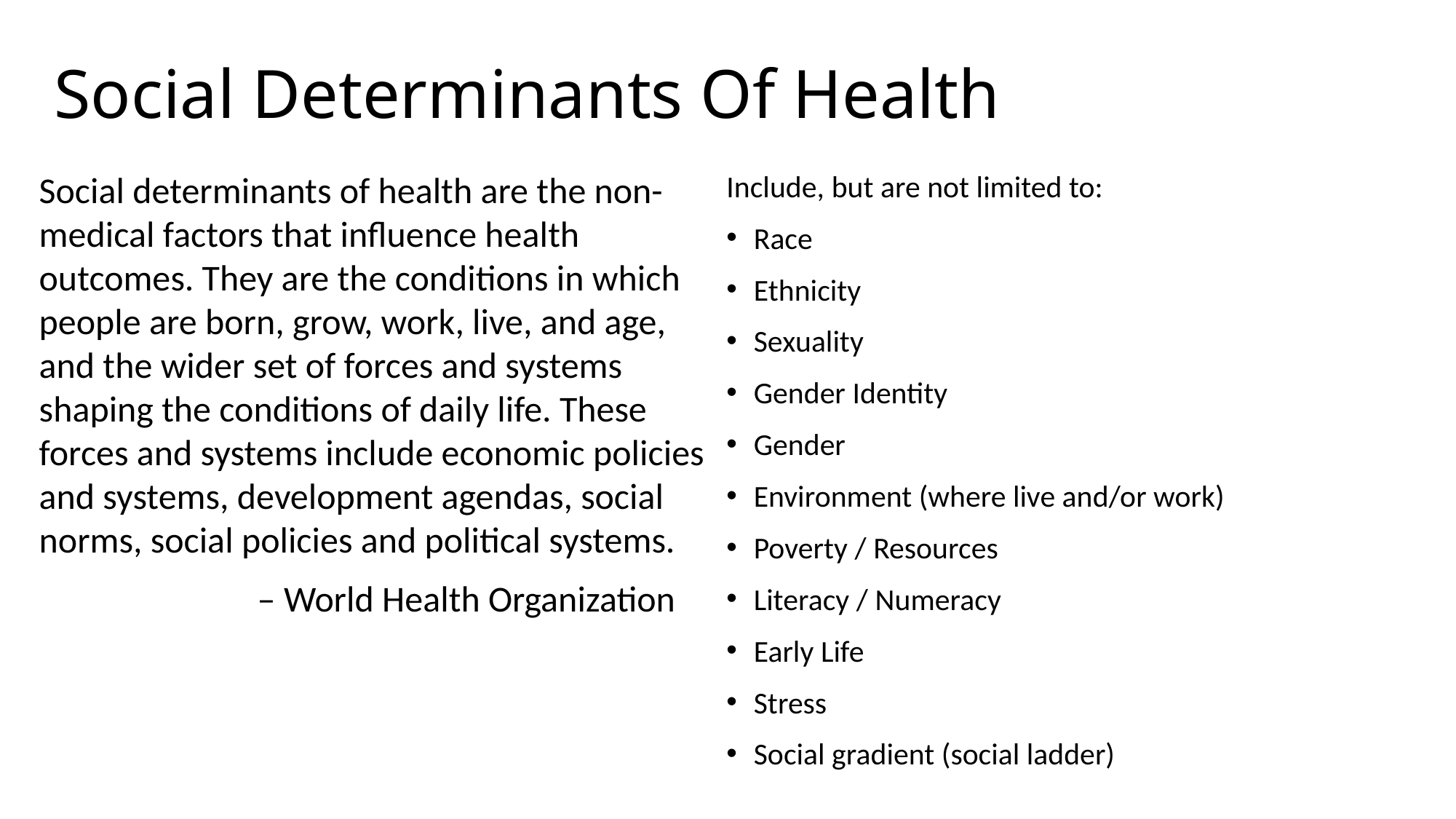

# Social Determinants Of Health
Social determinants of health are the non-medical factors that influence health outcomes. They are the conditions in which people are born, grow, work, live, and age, and the wider set of forces and systems shaping the conditions of daily life. These forces and systems include economic policies and systems, development agendas, social norms, social policies and political systems.
		– World Health Organization
Include, but are not limited to:
Race
Ethnicity
Sexuality
Gender Identity
Gender
Environment (where live and/or work)
Poverty / Resources
Literacy / Numeracy
Early Life
Stress
Social gradient (social ladder)

## Slide 10
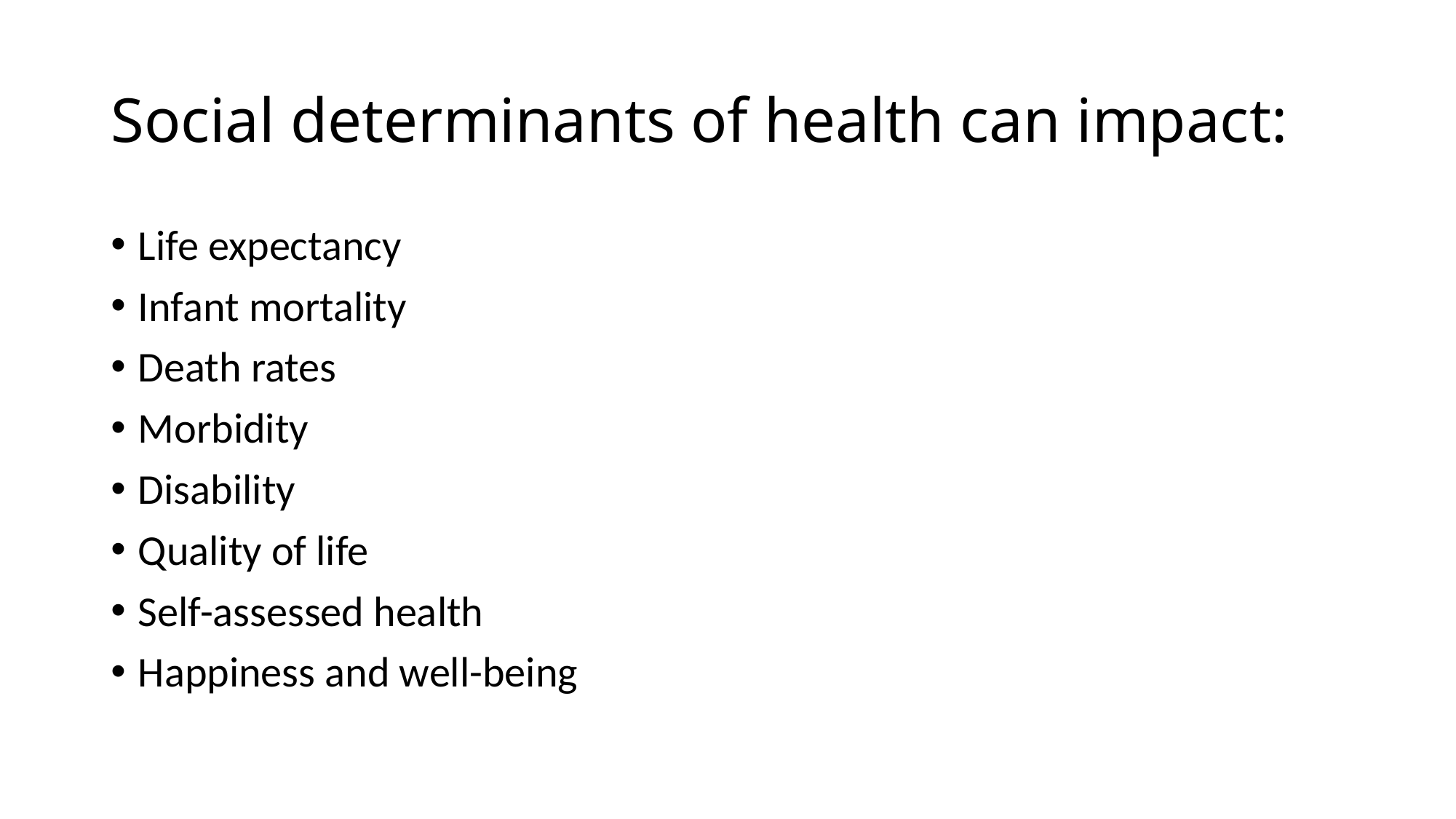

# Social determinants of health can impact:
Life expectancy
Infant mortality
Death rates
Morbidity
Disability
Quality of life
Self-assessed health
Happiness and well-being

## Slide 11
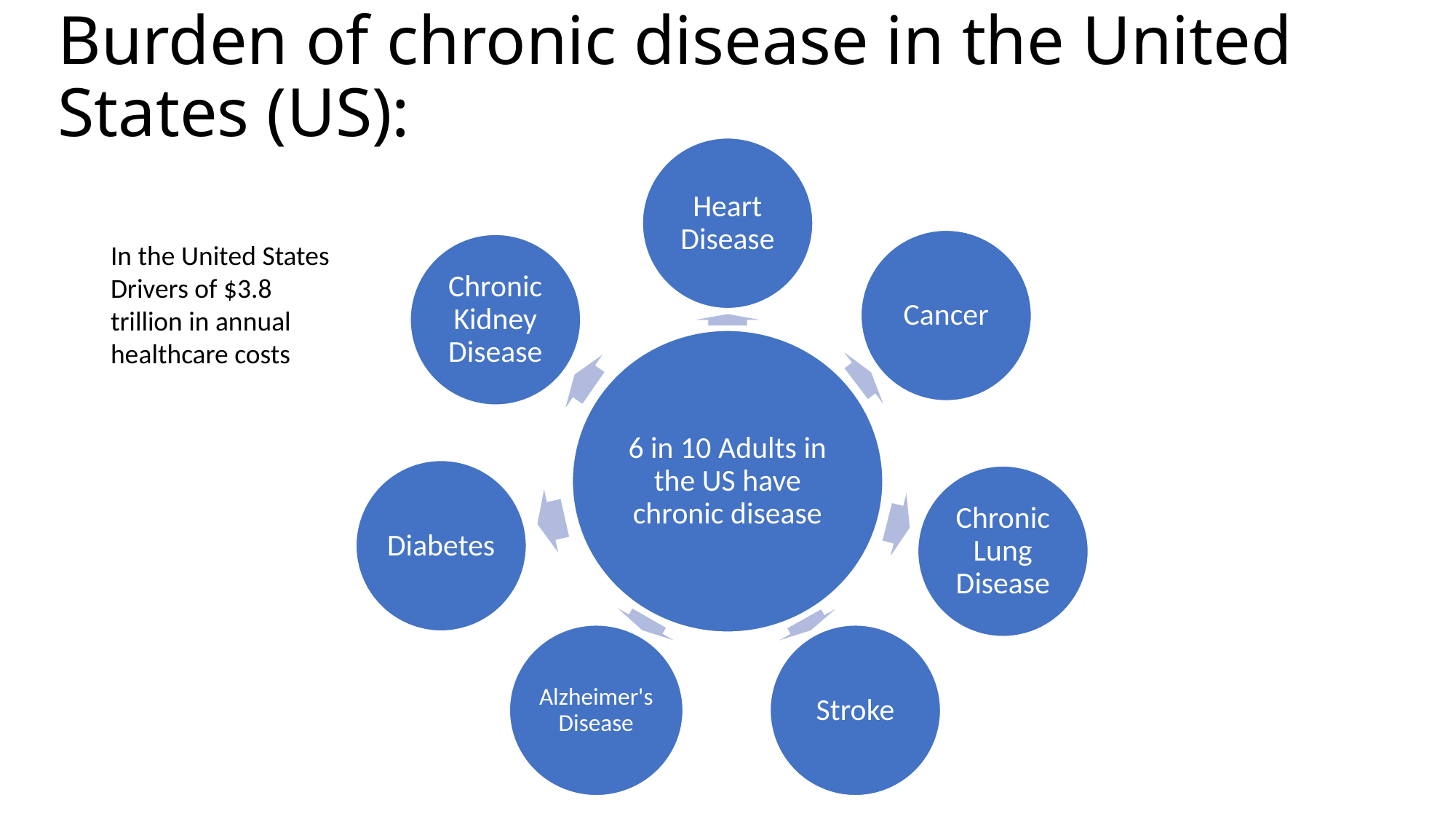

# Burden of chronic disease in the United States (US):
In the United States Drivers of $3.8 trillion in annual healthcare costs

## Slide 12
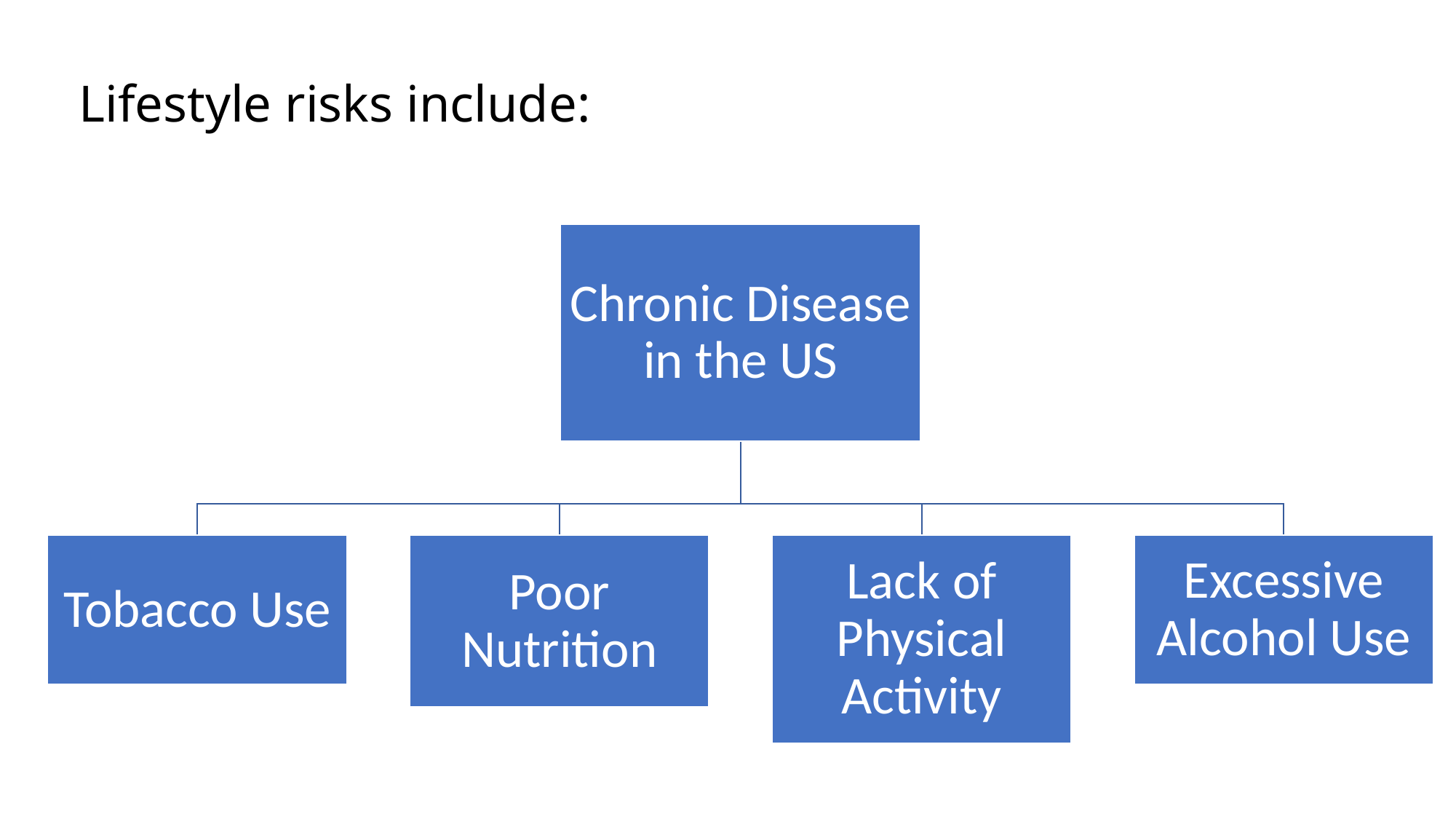

Lifestyle risks include:

## Slide 13
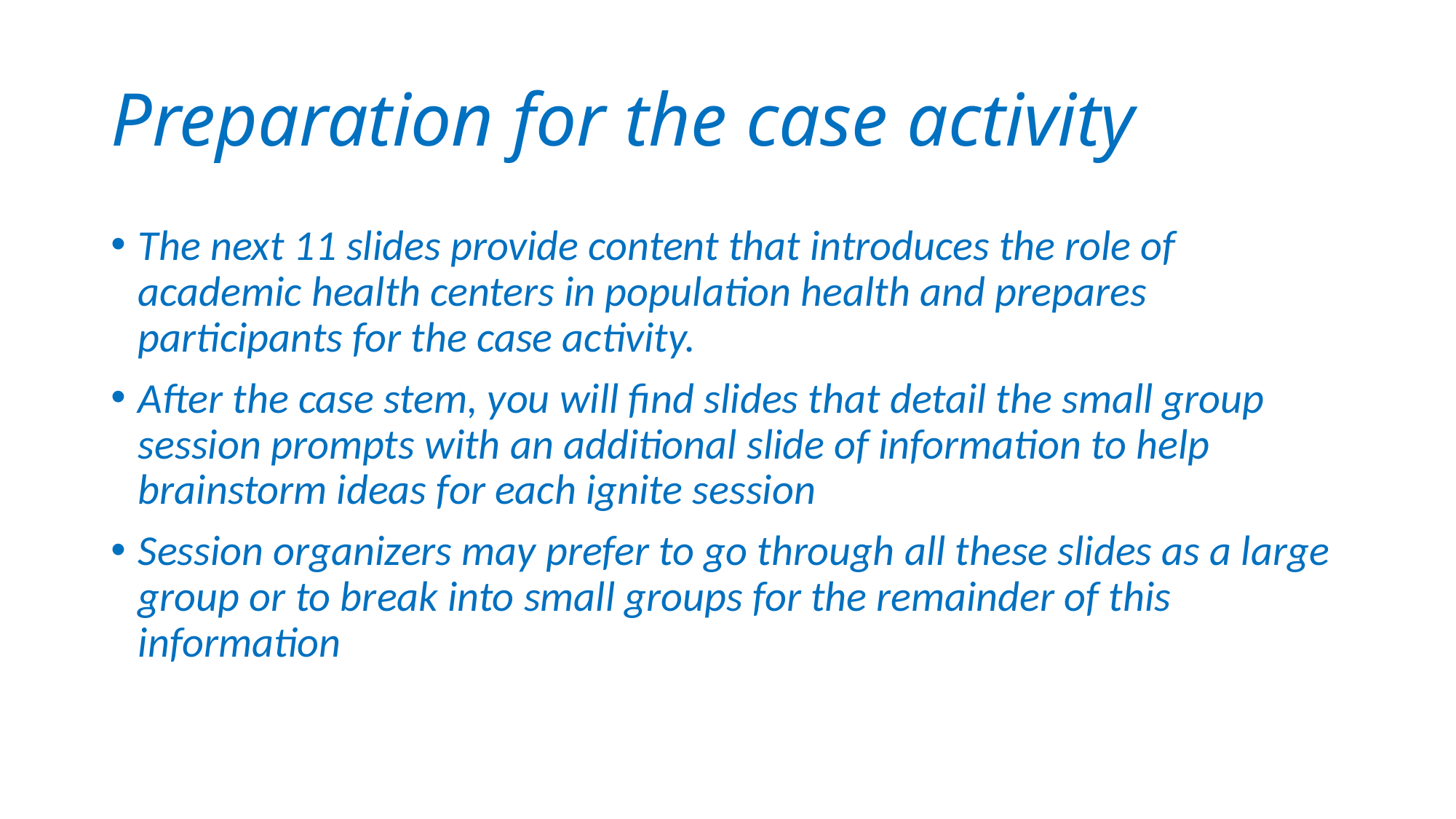

# Preparation for the case activity
The next 11 slides provide content that introduces the role of academic health centers in population health and prepares participants for the case activity.
After the case stem, you will find slides that detail the small group session prompts with an additional slide of information to help brainstorm ideas for each ignite session
Session organizers may prefer to go through all these slides as a large group or to break into small groups for the remainder of this information

## Slide 14
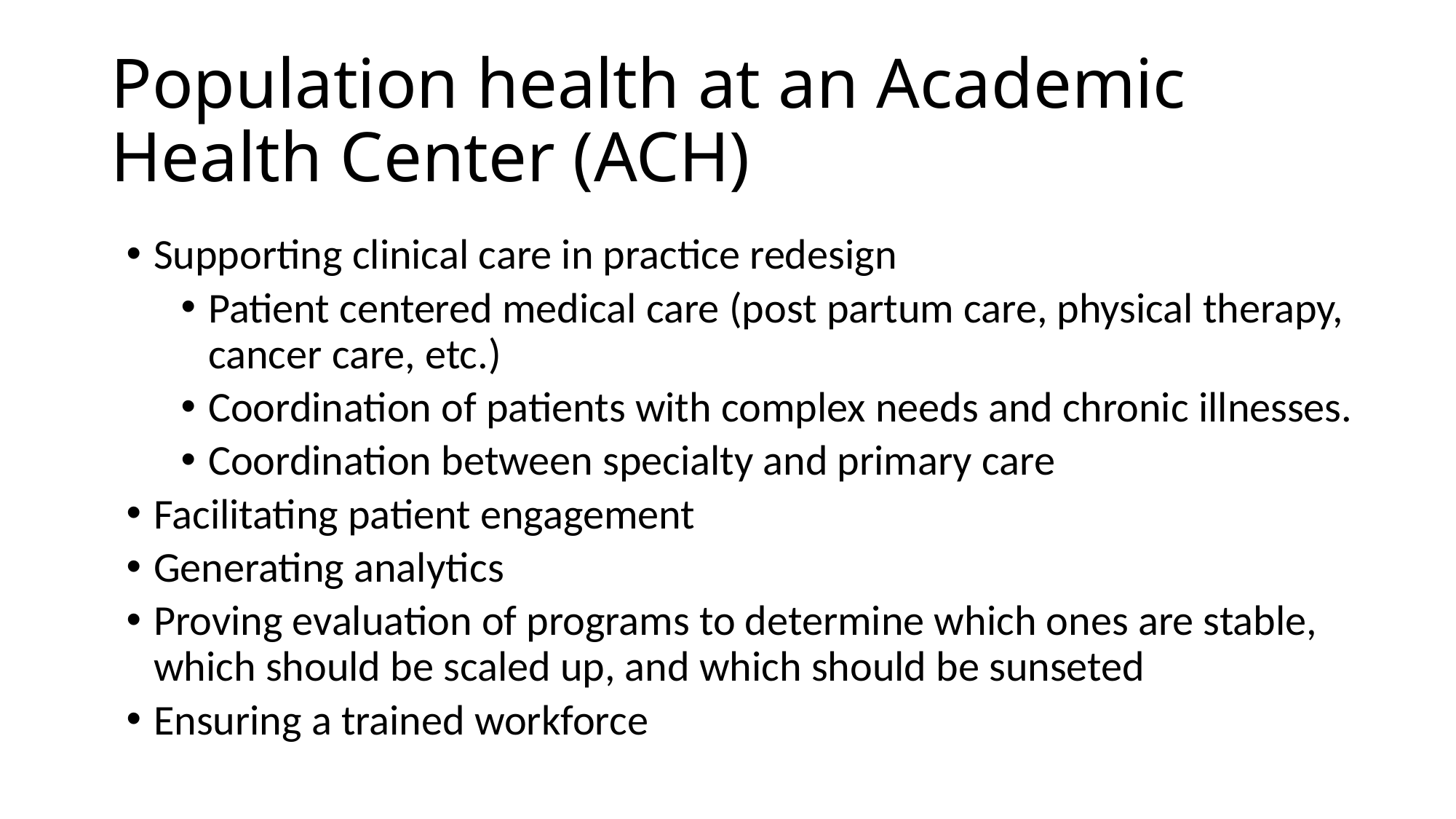

# Population health at an Academic Health Center (ACH)
Supporting clinical care in practice redesign
Patient centered medical care (post partum care, physical therapy, cancer care, etc.)
Coordination of patients with complex needs and chronic illnesses.
Coordination between specialty and primary care
Facilitating patient engagement
Generating analytics
Proving evaluation of programs to determine which ones are stable, which should be scaled up, and which should be sunseted
Ensuring a trained workforce

## Slide 15
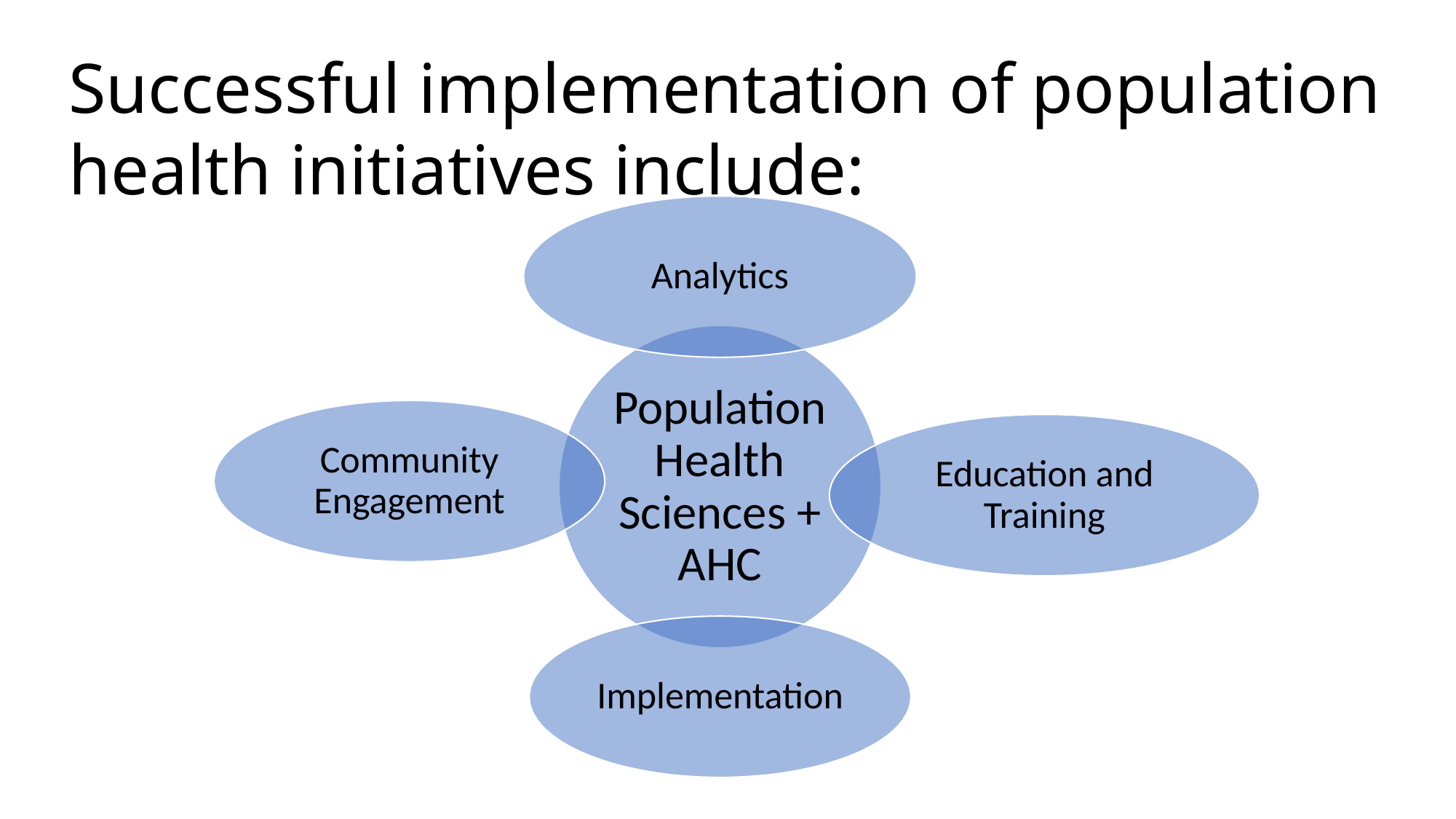

Successful implementation of population health initiatives include:

## Slide 16
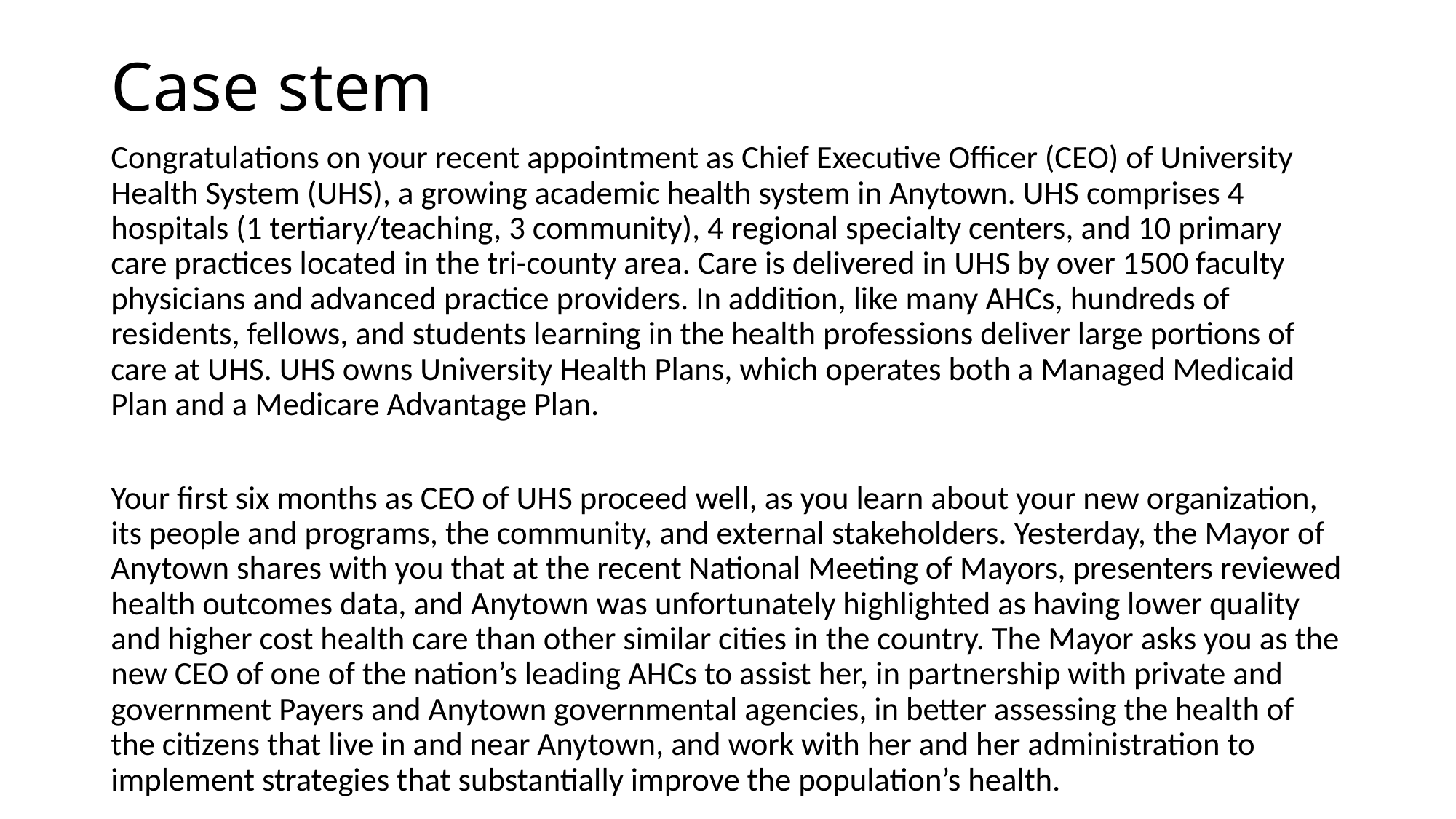

# Case stem
Congratulations on your recent appointment as Chief Executive Officer (CEO) of University Health System (UHS), a growing academic health system in Anytown. UHS comprises 4 hospitals (1 tertiary/teaching, 3 community), 4 regional specialty centers, and 10 primary care practices located in the tri-county area. Care is delivered in UHS by over 1500 faculty physicians and advanced practice providers. In addition, like many AHCs, hundreds of residents, fellows, and students learning in the health professions deliver large portions of care at UHS. UHS owns University Health Plans, which operates both a Managed Medicaid Plan and a Medicare Advantage Plan.
Your first six months as CEO of UHS proceed well, as you learn about your new organization, its people and programs, the community, and external stakeholders. Yesterday, the Mayor of Anytown shares with you that at the recent National Meeting of Mayors, presenters reviewed health outcomes data, and Anytown was unfortunately highlighted as having lower quality and higher cost health care than other similar cities in the country. The Mayor asks you as the new CEO of one of the nation’s leading AHCs to assist her, in partnership with private and government Payers and Anytown governmental agencies, in better assessing the health of the citizens that live in and near Anytown, and work with her and her administration to implement strategies that substantially improve the population’s health.

## Slide 17
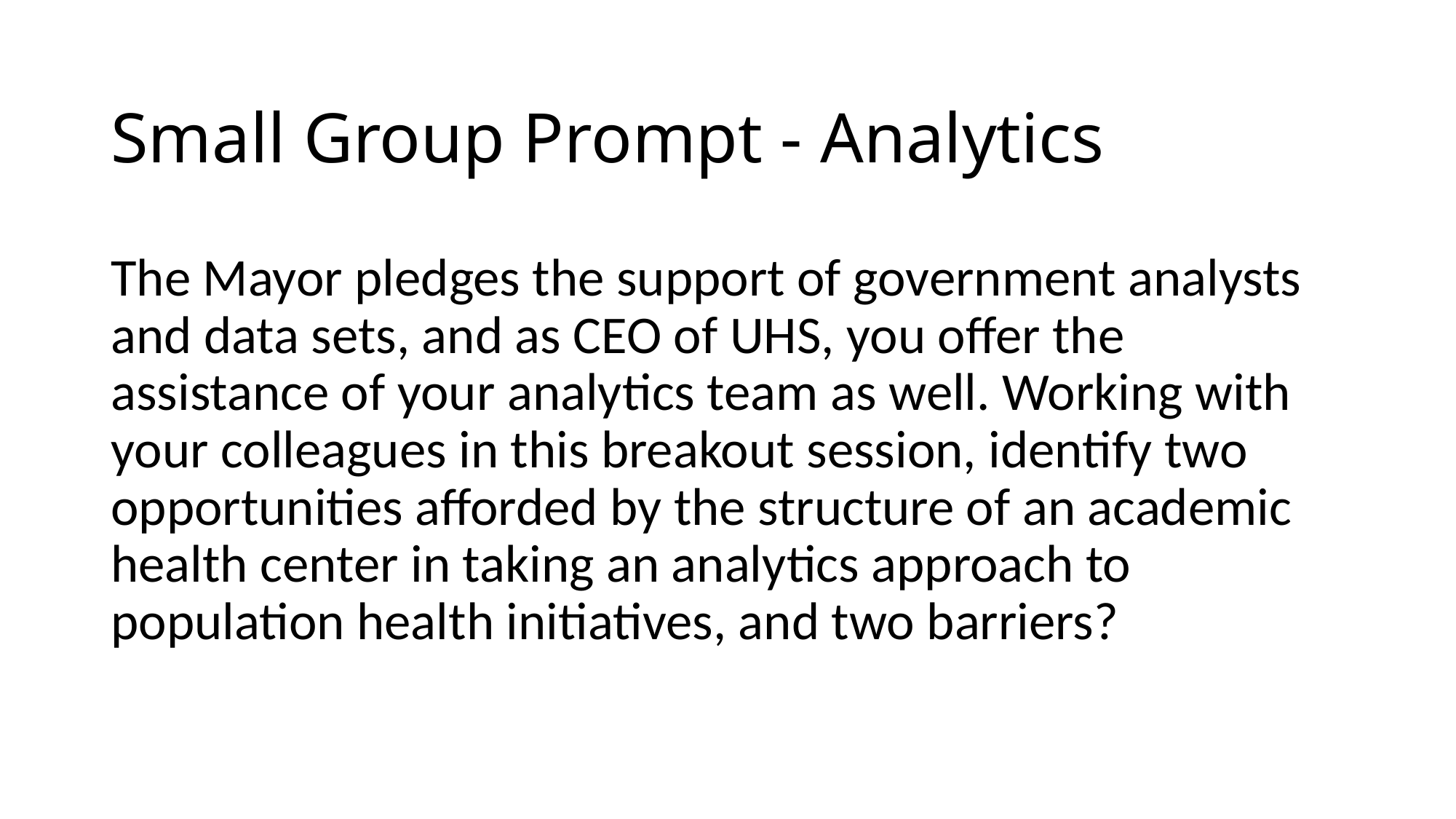

# Small Group Prompt - Analytics
The Mayor pledges the support of government analysts and data sets, and as CEO of UHS, you offer the assistance of your analytics team as well. Working with your colleagues in this breakout session, identify two opportunities afforded by the structure of an academic health center in taking an analytics approach to population health initiatives, and two barriers?

## Slide 18
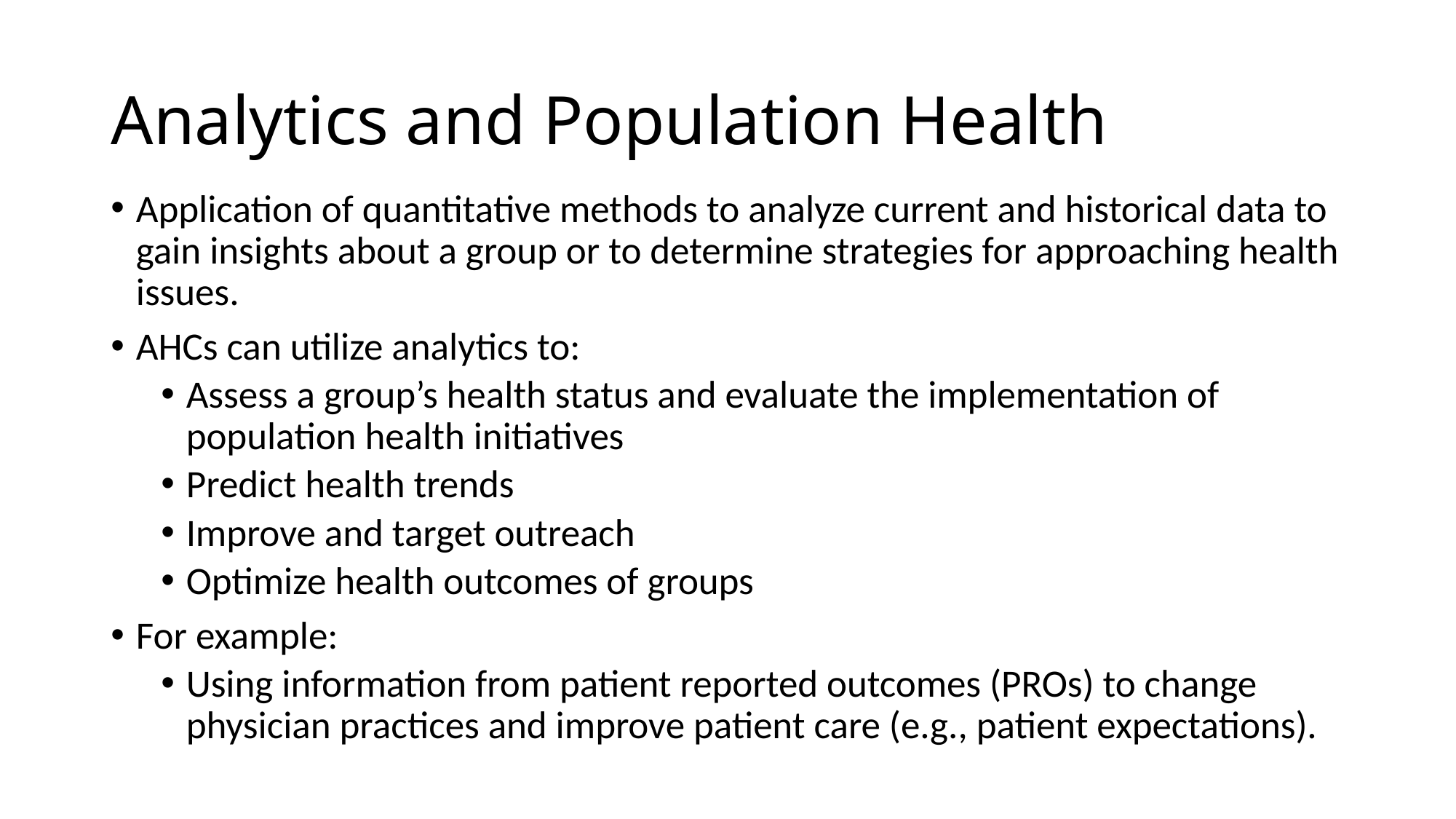

# Analytics and Population Health
Application of quantitative methods to analyze current and historical data to gain insights about a group or to determine strategies for approaching health issues.
AHCs can utilize analytics to:
Assess a group’s health status and evaluate the implementation of population health initiatives
Predict health trends
Improve and target outreach
Optimize health outcomes of groups
For example:
Using information from patient reported outcomes (PROs) to change physician practices and improve patient care (e.g., patient expectations).

## Slide 19
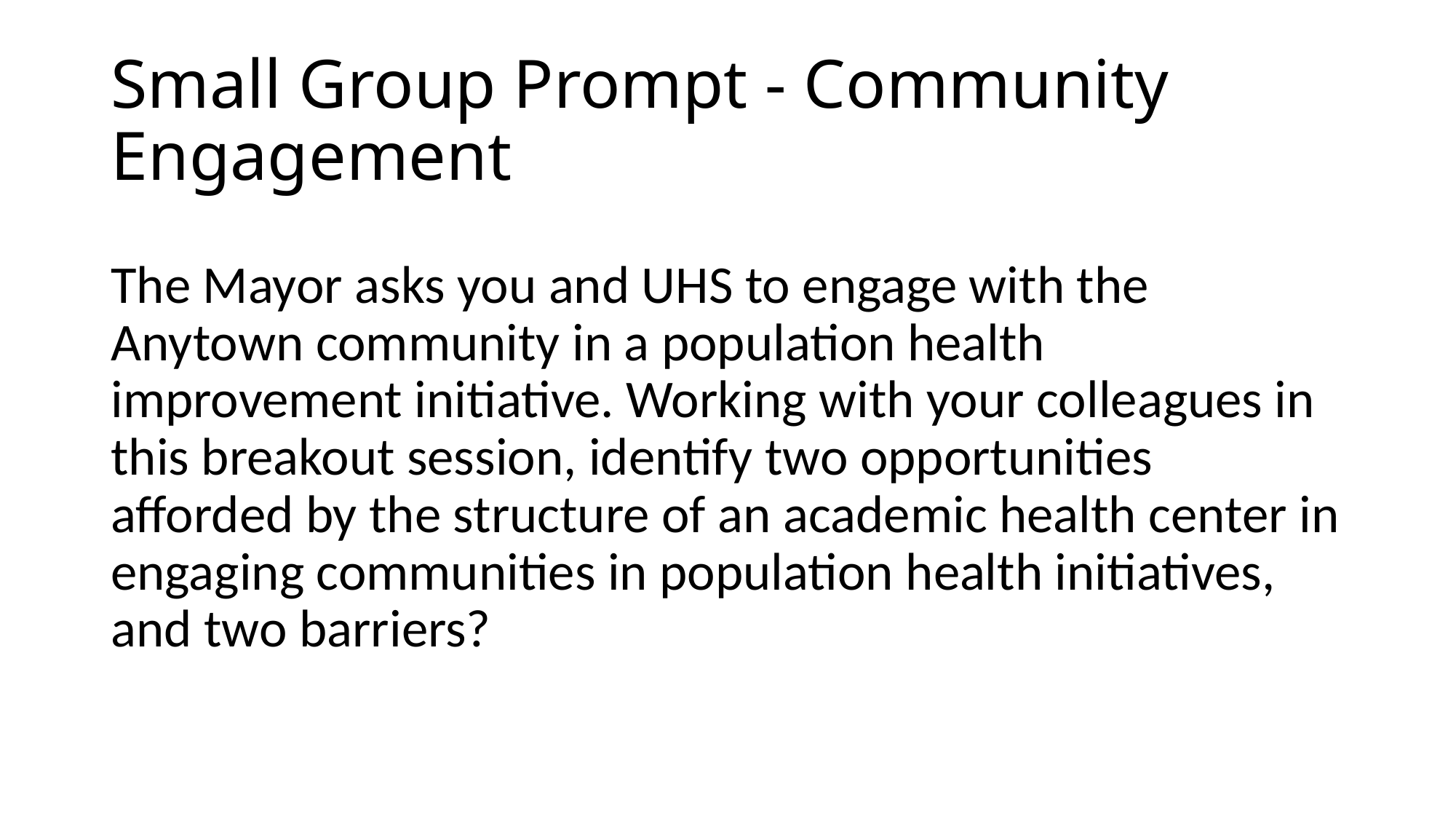

# Small Group Prompt - Community Engagement
The Mayor asks you and UHS to engage with the Anytown community in a population health improvement initiative. Working with your colleagues in this breakout session, identify two opportunities afforded by the structure of an academic health center in engaging communities in population health initiatives, and two barriers?

## Slide 20
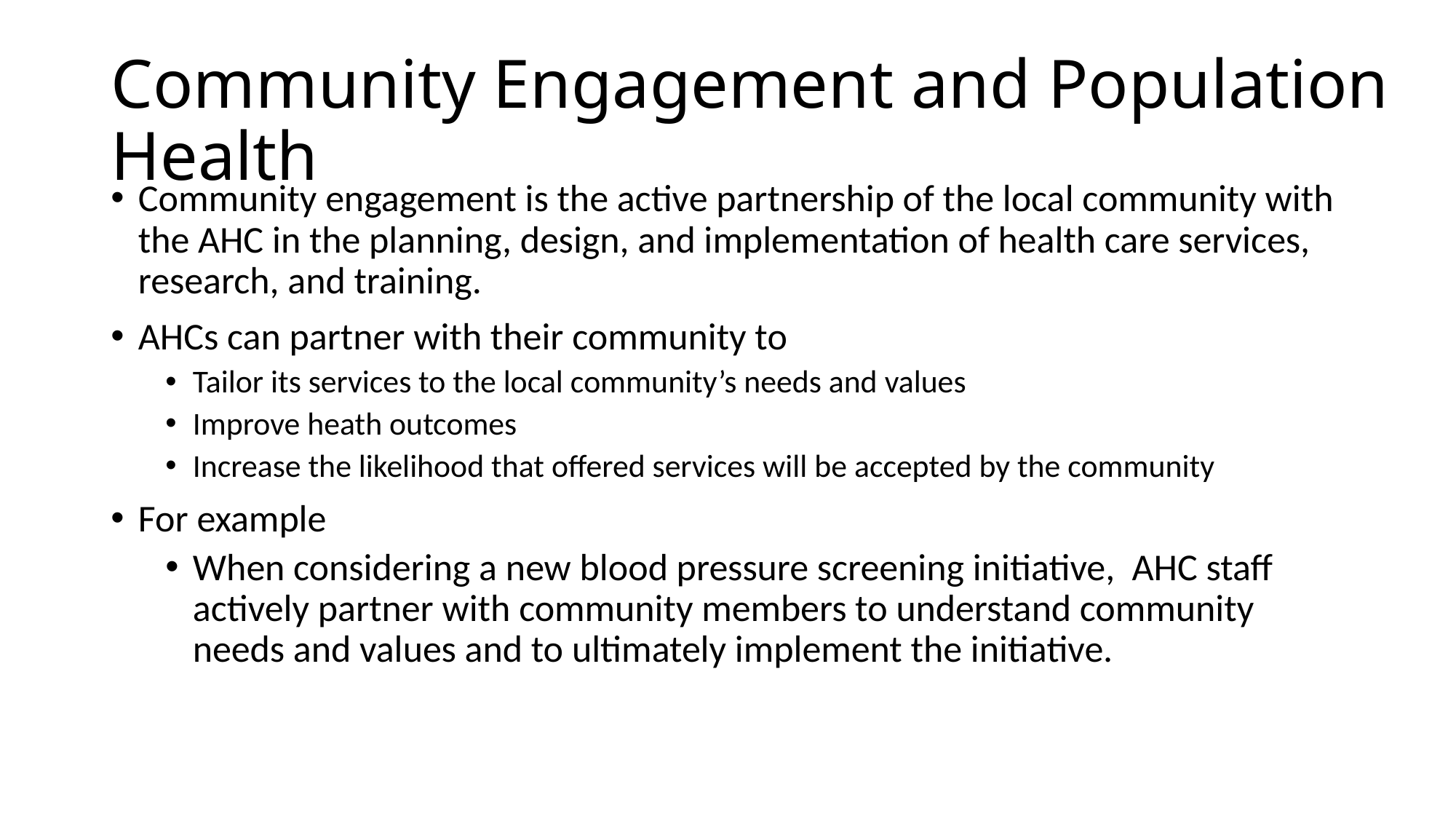

# Community Engagement and Population Health
Community engagement is the active partnership of the local community with the AHC in the planning, design, and implementation of health care services, research, and training.
AHCs can partner with their community to
Tailor its services to the local community’s needs and values
Improve heath outcomes
Increase the likelihood that offered services will be accepted by the community
For example
When considering a new blood pressure screening initiative, AHC staff actively partner with community members to understand community needs and values and to ultimately implement the initiative.

## Slide 21
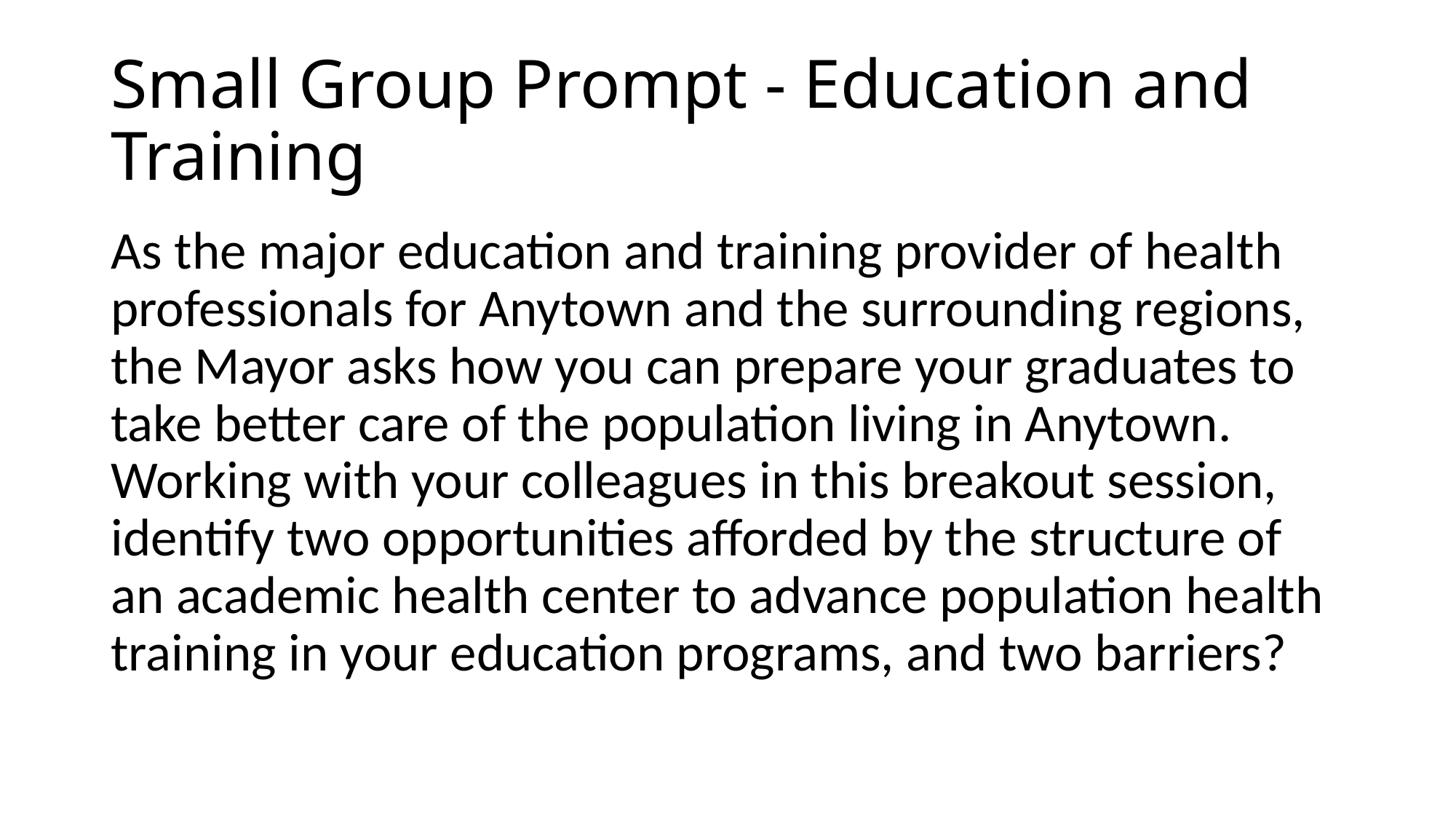

# Small Group Prompt - Education and Training
As the major education and training provider of health professionals for Anytown and the surrounding regions, the Mayor asks how you can prepare your graduates to take better care of the population living in Anytown. Working with your colleagues in this breakout session, identify two opportunities afforded by the structure of an academic health center to advance population health training in your education programs, and two barriers?

## Slide 22
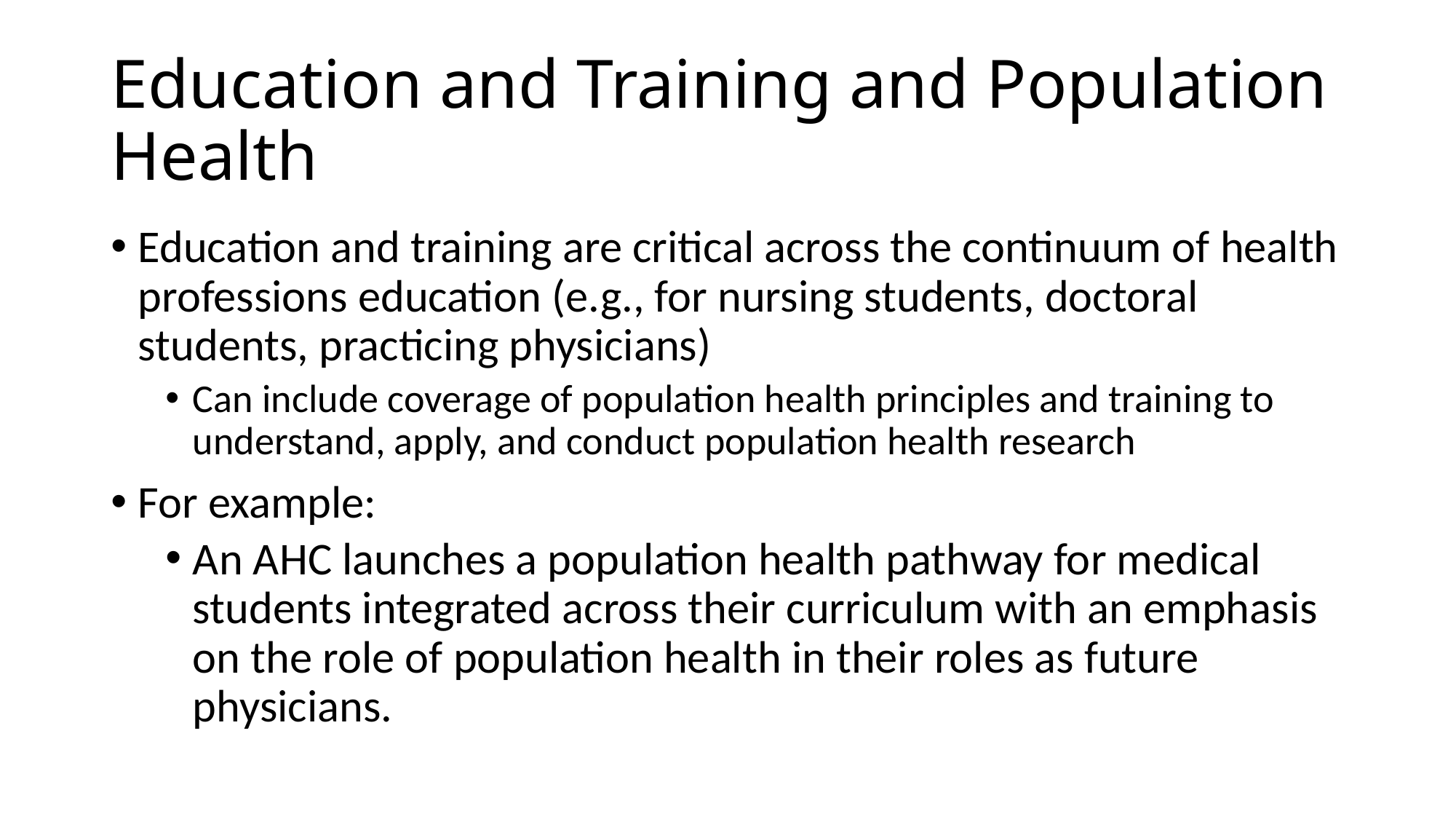

# Education and Training and Population Health
Education and training are critical across the continuum of health professions education (e.g., for nursing students, doctoral students, practicing physicians)
Can include coverage of population health principles and training to understand, apply, and conduct population health research
For example:
An AHC launches a population health pathway for medical students integrated across their curriculum with an emphasis on the role of population health in their roles as future physicians.

## Slide 23
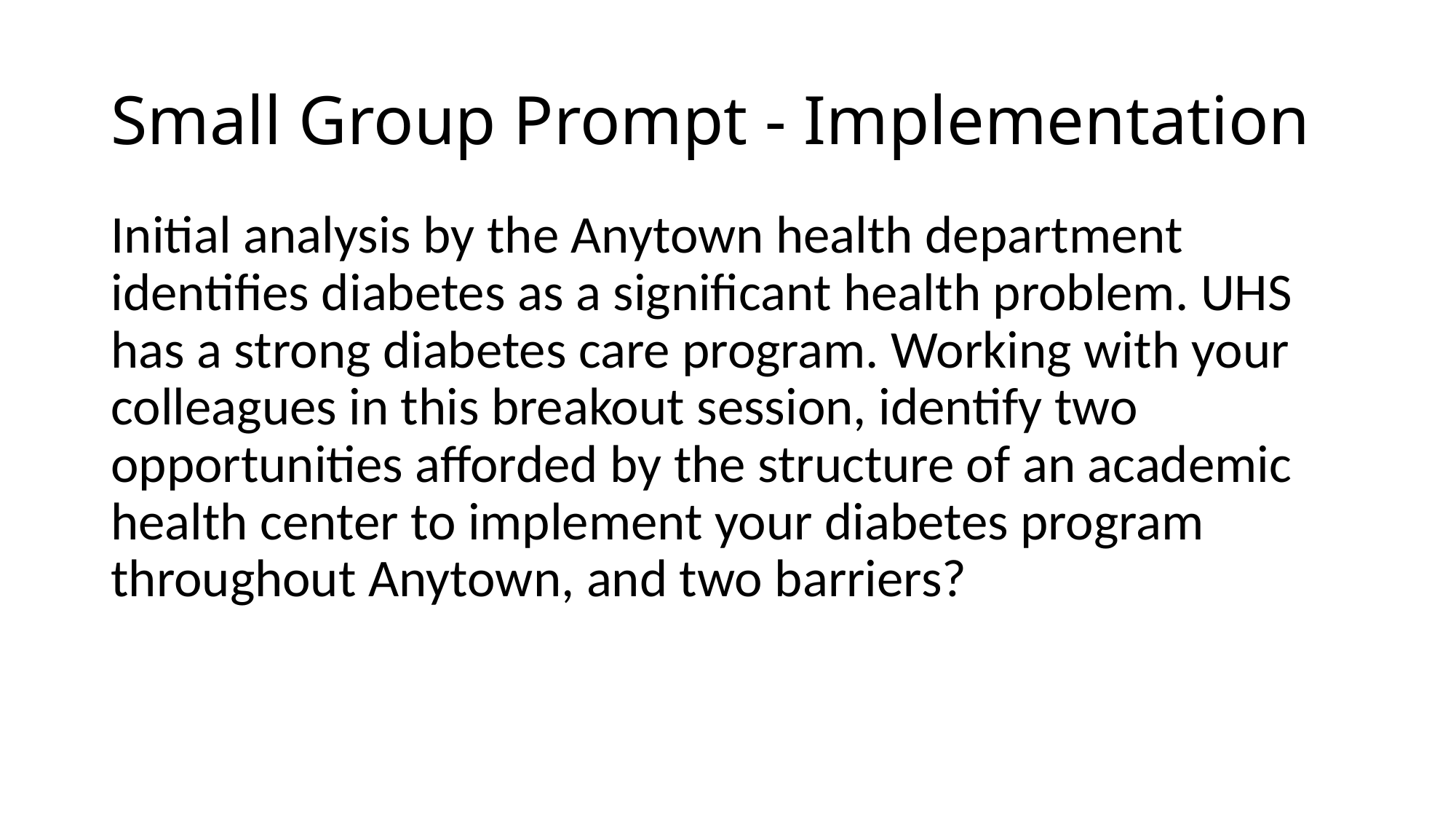

# Small Group Prompt - Implementation
Initial analysis by the Anytown health department identifies diabetes as a significant health problem. UHS has a strong diabetes care program. Working with your colleagues in this breakout session, identify two opportunities afforded by the structure of an academic health center to implement your diabetes program throughout Anytown, and two barriers?

## Slide 24
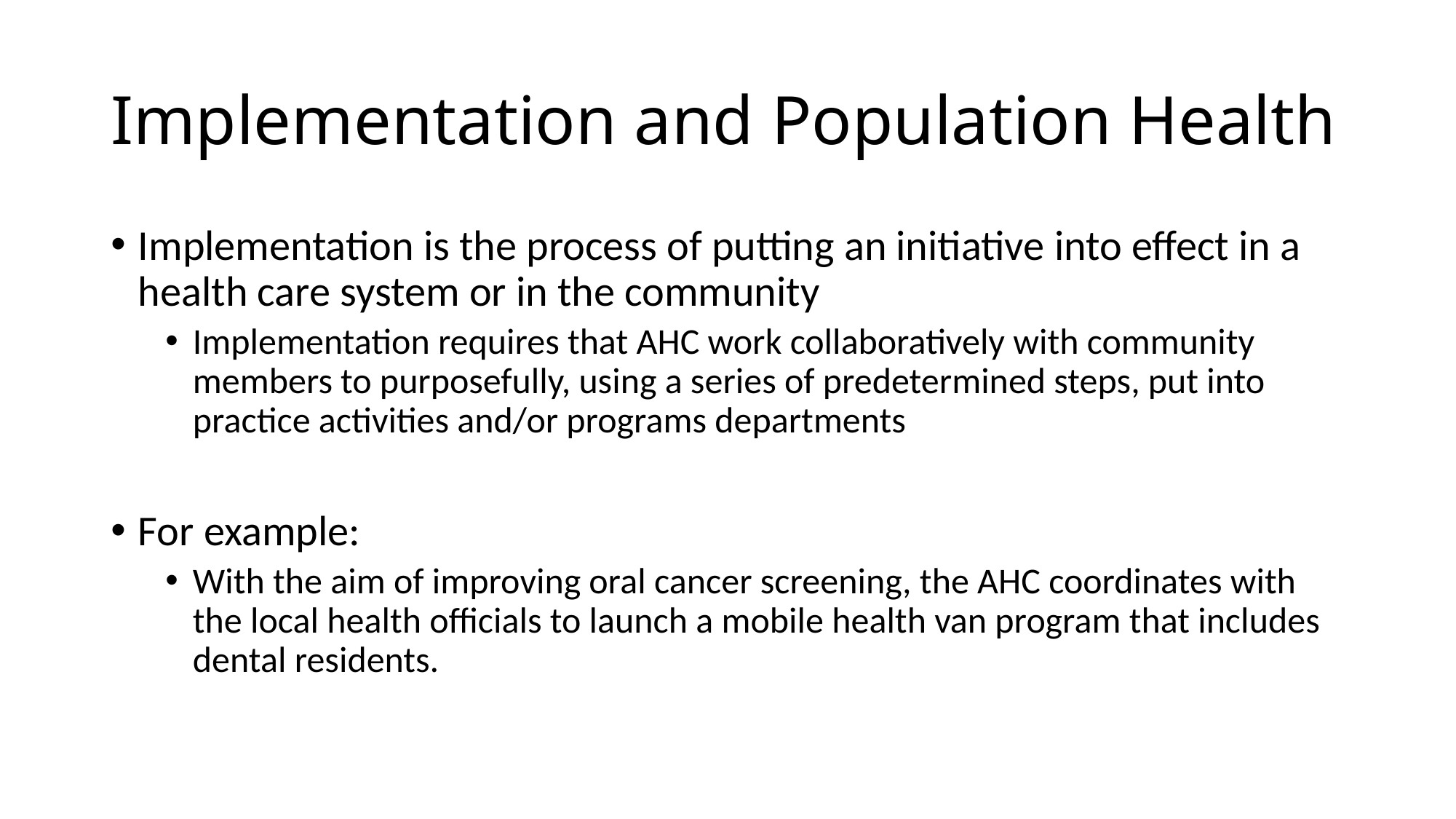

# Implementation and Population Health
Implementation is the process of putting an initiative into effect in a health care system or in the community
Implementation requires that AHC work collaboratively with community members to purposefully, using a series of predetermined steps, put into practice activities and/or programs departments
For example:
With the aim of improving oral cancer screening, the AHC coordinates with the local health officials to launch a mobile health van program that includes dental residents.
