# Supplementary material for: Population Health at the Academic Health Center: An Interactive, Multipart, Case-Based Session for Executives, Faculty, and Administrators
Source: MedEdPORTAL. 2022 Jan 7;18:11204. doi: 10.15766/mep_2374-8265.11204 (PMC8738160; doi:10.15766/mep_2374-8265.11204)
Supplement: Supplementary file 1 — Call for Abstracts.docxReviewer Rubric.docxCase Stem and Small-Group Prompts.docxSession Evaluation.docxIntroduction to Population Health.pptxFacilitator Guide.docx [file mep_2374-8265.11204-s001.zip › F. Facilitator Guide.docx]

Facilitator’s Guide:

In October 2020, a virtual interactive multi-part, case-based session was offered at the Association of Academic Health Centers’ (AAHC) annual meeting. The 90-minute session was targeted at academic health center (AHC) executives, faculty and administrators to provide insight into how population health initiatives are conducted at AHCs and to discuss how the AHC structure can be leveraged to promote population health.

To assist instructors in implementing this session at their own AHC, we created this facilitator guide, which provides additional logistical details for conducting the session and, in some cases, potential alternative approaches to delivering the content. We have also provided reflections on our offering of the training, however, we encourage instructors to be flexible in their approach such that they consider their specific audience and context and modify as needed.

This guide is divided into three components to provide further guidance on:

- the ignite-style presentations;
- the case-based activity; and
- population health background information as addressed in an optional PowerPoint slide deck.

Of note, while the components are represented in the above order, instructors are free to rearrange the sequence of components.

**Ignite-style session**

Ignite-style presentations (<http://www.ignitetalks.io/>) are rapid-paced presentations conducted in five minutes or less with a limited amount of PowerPoint slides that are advanced automatically usually in 20 second intervals. In our instance, seven presenters were limited to five PowerPoint slides, together accounting for 35 minutes of the overall session. The number of presenters and time dedicated to this component could be modified. However, we would encourage limiting presentations to five minutes or less to ensure that the flow of the session remains fast paced. Unlike a traditional ignite presentation, participants in our session were permitted to use the allotted time as they liked without a timed sequential forwarding of slides. However, the number of slides allowed and whether or not to automatically advance the slides could be modified. Additionally, depending on the time allotted to the ignite session, instructors could consider allowing an additional 2-3 minutes for follow-up questions to each presenter or provide time for questions after all presentations have occurred.

The goal of these short presentations was to provide attendees with brief glimpses into population health initiatives that had been implemented at the presenter’s AHC. In our session, we invited conference attendees to submit brief abstracts, which were then judged for inclusion. See Appendix A for the call for proposals. For examples of abstracts, please see the AAHC website which features all abstracts submitted to our call <https://www.aahcdc.org/Initiatives/Presidents-Councils/AAHC-Presidents-Council-on-Health-Plans-and-Population>. This juried approach could be replicated or instructors may choose to directly invite presenters from their AHC. If the instructors are unable to attract presenters they might consider reviewing the literature to identify interesting initiatives and then invite those authors to present either virtually or in-person. Additionally, the instructors could reach out to related associations such as the Interdisciplinary Association for Population Health Science (iaphs.org) or the American Public Health Association (<https://www.apha.org/>) for suggested presenters.

The session was opened by an AHC CEO with a brief introduction to the goals of the session and to the format of ignite presentations. This individual served as an “emcee” throughout the entire session. Although we opened with these presentations they could take place at any point during the overall session. Additionally, ignite presentations can be held virtually or in-person. In either setting, due to the tight timeframe of the presentations, we recommend that participant presentation slides be collated into a single slide deck to be presented by one individual. This will limit time spent transitioning between presenters and ensure that participants do not exceed the number of permitted slides.

Logistics for Ignite-style presentations

- Time: 35 minutes
  - This may be adjusted depending on the number of speakers who will each have 5 minutes for their presentation
- Resources:
  - A single PowerPoint slide deck that includes the presentations from all speakers, including any introductory slides for the session
  - A timing device

|  | Facilitator Role | General Tips |
| --- | --- | --- |
| **Pre-session** |  |  |
| At least 8 weeks prior to session | Identify and invite speakers to participate directly or to submit an abstract for consideration (See Appendix A for a call for abstracts) | Invitations must clearly describe the presentation’s brief format. |
| At least 6 weeks prior to the session | If using an abstract approach, recruit reviewers and provide guidance for reviewing (See Appendix B for a review scoring form) | Consider setting up an online form/survey (e.g., GoogleForm or SurveyMonkey Survey) to capture and collate reviewer responses |
| At least 4 weeks prior to the session | If using the abstract selection approach, notify selected presenters | The notification must include clear presentation instructions and deadlines for submission of slides |
| One week prior to the session | Collect all speaker slides and assemble a single slide deck | Facilitators may wish to include introductory slides to familiarize attendees with the nature of the session |
| **Day of the session** |  |  |
| 20 minutes before the session | Gather all presenters for a check of technology, quick review of slides and an introduction to the timer that will be used | Remind session participants of the time limit |
| 5 minutes | Facilitator introduces the session, including a brief description of the session format | Project a single slide deck controlled by the facilitator |
| 30 minutes | Presenters each present their five slides in five minutes or less | Allow only one individual presenter per initiative  Engage a timekeeper to ensure participants stay on time  Encourage presenters to not only focus on the successes, but also lessons learned and surprises from their initiatives  Have presenters briefly introduce themselves to save time |
|  | Briefly close the ignite session and thank participants. If this is the end of all activities, ask the participants to complete a brief evaluation of the session (See Appendix D) | Prepare to send out follow-up emails to remind participants to complete the evaluation |

**Case-based activity**

We next engaged our participants in a case-based activity, which was allotted 45 minutes. This activity included both small and large group interactions, which were each 20 minutes in duration. Those seeking to implement this activity might consider expanding the overall timeframe. If extra time is available, we would encourage expanding the small group component as logistically it can take time for participants to assemble. We discourage shortening this activity as it would be difficult for the participants to engage with the case and their groups in such a limited amount of time.

Upon registration for the session, which took place several weeks earlier, participants self-selected their small groups based on four topics: analytics, community engagement, education and training and implementation. Small group topics were determined by the planning committee. These topics could be modified to meet the needs and expertise of those implementing the training. Participants were not provided information about the topic during the selection process and were asked to select the topic of most interest to them. We would encourage providing participants at registration brief descriptions of the topics to facilitate their group selection. It would also be possible to assign participants to topics based on their roles, level of knowledge, training background, etc. Additionally, participants could also be directed to self-select based on these or other characteristics. In our session, participants attended only one small group and were unable to freely switch between groups. Depending on time available, organizers could run more than one iteration of small groups so that participants could engage with multiple topics.

In our session, small groups ranged from 7-12 participants. We did not find that having groups of varying sizes impacted the activity. To facilitate participation, we attempted to keep the groups somewhat small. This required that we monitor registration to ensure that no group became too large, which based on the AAHC’s experience with online small groups was considered over 15 participants. This resulted in the need to close registration for one of the topics. So as not to disappoint registrants, we would encourage organizers to consider asking participants to indicate a second-choice topic or if a topic proves particularly popular to create a second group for that popular topic.

To start the small group component, the emcee briefly introduced the activity, including reading the case while the participants were still in the large group, and alerting them that they would be automatically transferred to their small groups using Zoom’s small group functionality. If a session is to be conducted online, we encourage organizers to consider the capabilities of their online platform as it is critical that the transition be smooth to avoid participant frustration and not waste time.

Small groups were facilitated by an AHC executive and a member of the professional special interest group: Population Health Leaders in Academic Medicine (PHLAM). We contacted PHLAM leadership several months prior to the session with an invitation to participate. Organizers could consider recruiting facilitators from a professional group, such as PHLAM, but we also would encourage them to reach out to their population health faculty and AHC administrators. Facilitators did not receive training for the session, but were provided with the case and our expectations for their role several weeks prior via email. In the email, we reassured the facilitators that they did not need to prepare any materials. Instead, we asked the facilitators to acts as group participants sharing their thoughts and experiences with their topic. We also asked that the facilitators run the logistics of the session and as needed guide the participants back to the task at hand if they strayed off topic. As a component of their duties, the facilitators asked for volunteers to act as the group’s scribe and reporter. The reporter was tasked with reporting back to the large group.

The day before the session, we emailed participants the case such that they could prepare for the session if they so desired. We provide the case stem and small group prompts in Appendix C. We are pleased for others to use our case materials, but also encourage users to tailor them to align them with their own specific goals and contexts.

Once participants were in their small groups, they were welcomed by their two facilitators and the group began sharing brief introductions that included their name, role and institution. Depending on the time available and group size, introductions could be expanded to further build group rapport. Next the facilitators asked for volunteers to act as a scribe and a reporter. During the small groups, the Zoom screen featured participants’ video, the case, and the prompts for the group’s topic. The prompt asked participants to brainstorm two opportunities and two barriers created by the structure of an AHC. We experienced across groups that participants joined into the conversation naturally with some participants simply speaking out and other using Zoom’s raise hand feature. If this had not happened, facilitators were asked to volunteer their thoughts briefly to stimulate discussion. After 20 minutes of discussion, participants were returned to the large group using Zoom’s functionality.

Participants were welcomed back to the session by the emcee. The reporters from each group were then invited to report to the group with each reporter provided 2-3 minutes. Once all groups had reported, the emcee asked the entire group to discuss ways in which the groups’ reports overlapped. After 20 minutes, the emcee closed the session by thanking the participants for their time and participation and encouraged them to continue discussing this topic with their colleagues upon returning to their AHC.

Logistics for Case-based activity

- Time: 40 minutes
  - 20 minutes for the small group activity
  - 20 minutes for the large group debrief activity
- Resources:
  - Case stem and case prompt (Appendix C)

|  | Facilitator Role | General Tips |
| --- | --- | --- |
| **Pre-session** |  |  |
| At least 6 weeks prior to session | Identify and invite small group facilitators. Provide the facilitators with the case stem and prompts. | Consider inviting population health faculty from your institution or contacting a professional association like the Interdisciplinary Association of Population Health Science |
| At least 4 weeks prior to the session | Invite participants to register for the session and to indicate their preferred small group (analytics, community engagement, education and training, and implementation) |  |
| The day before the session | Email participants a reminder of the session with the case stem | Do not count on that participants will have read the case before hand |
| **Day of the session** |  |  |
| 20 minutes before the session | Gather all small group facilitators for a check of technology and to address any questions or concerns. Remind them that they are facilitating not teaching the small group. | Troubleshoot your online platform, especially in relation to its ability to transfer facilitator participants in and out of small groups |
| 5 minutes | Briefly introduce the session, including a brief description of the session format |  |
| 20 minutes  Small Group Activity | Send participants to their small groups for discussion of the case and prompts  Small group facilitators briefly introduce the task and identify a volunteer scribe to take notes and a reporter to report back to the large group | Provide small group members with a two-minute warning prior to the end of the small group session  If participants are uncomfortable speaking out in the group they can be encouraged to also use the online platforms chat feature |
| 20 minutes  Large Group Activity | Participants return to the large group  Small group reporters are invited to present their group’s discussion in 2-3 minutes | Allow all group reporters to present prior to allowing general questions and comments |
|  | Briefly close the ignite session and thank participants. If this is the end of all activities, ask the participants to complete a brief evaluation of the session (See Appendix D) | Prepare to send out follow-up emails to remind participants to complete the evaluation |

**Population health background information**

We believe our session could be implemented with a variety of audiences, which may include participants with varying levels of familiarity with population health. Thus, we created a supplemental PowerPoint presentation (Appendix E) that provides background information on population health and additional scaffolding for the case-based activity. We would encourage those that choose to use this supplemental resource to consider extending their session by at least 15-30 minutes.

Prior to presenting the PowerPoint, we would encourage organizers to review the slide deck, which includes 23 slides, to identify those slides that would be of value to their audience. Organizers should feel free to be creative with the organization and content of the slides to meet their objectives for the session and the needs of their audience. They may also decide that the PowerPoint is unnecessary for their session. We have attempted to make the slide deck modular, such that organizers may choose to implement specific sections or present it in its entirety. Additionally, participants could consider presenting the slides at various points in their session. For example, it would be possible to open the session with the slide deck or to present it prior to launching the case-based activity. To ensure relevance, we do not recommend featuring the slides after the case-based activity.

In the slide deck, we provide talking points and suggestions for presentation in the notes section of many of the slides. For example, we have noted in slides 3-6 that organizers could consider presenting these at the beginning of either the ignite presentations or case-based activity to provide overall context for participants. We have also included several slides that provide suggested guidance for presentation. These slides are in blue font and should be removed from the deck prior to presentation. Below for each slide we provide the text of the slide and any notes included in its notes section.

Logistics

- Time: 15-30 minutes
- Resources:
  - Slide deck (Appendix E)

|  | Facilitator Role | General Tips |
| --- | --- | --- |
| **Pre-session** |  |  |
| At least two weeks prior to the session | Determine if the supplemental slides will be integrated. If yes, consider if when in the session the slides will be presented | Ensure that additional time is added to the overall event to allow for the addition of the slide presentation |
| At least a week prior to the session | Email participants the slide deck | Do not count on participants having reviewed the slides ahead of time |
| **Day of the session** |  |  |
| 5 minutes | Briefly introduce the slide presentation session | Reference that the slide content will facilitate their understanding of the next sections of the session |
| 20 minutes | Present the power point slides and related notes | Consider embedding local examples of population health initiatives if they exist |
|  | Briefly close the slide portion and segue to either the ignite-style session or case-based activity |  |

Overview of slide deck

Slide 1: A brief orientation to using this slide deck and facilitator guide to support the session: Population Health at the Academic Health Center

- We created this educational session with the intent of implementing it in a variety of settings. We recognize that participants in each setting may have varying levels of familiarity with population health. Thus, we created the accompanying PowerPoint deck to provide background on population health and related scaffolding for the included session activities.
  - We encourage instructors to be creative with this deck’s content using it as building blocks to develop their own presentations.
- Throughout the PowerPoint we include slides, indicated by use of blue font, which provide suggestions for how you might utilize the presented context. We suggest removing these prior to presentation.
- Throughout this Facilitator Guide we include blue font that is intended to be suggestions for how to use the guide.
- We have attempted to make this content modular such that it can be used in its entirety or instructors can select specific components to suit their needs.

Slide 2: Population Health 101

- The next 4 slides provide content that introduces population health and its core components.
  - Session organizers might consider providing these slides to participants prior to the session or at the beginning of the session to orient participants to the topic.
  - Session organizers might consider removing these slides if participants are already familiar with population health.

Slide 4: Goal of Population Health

- The goal of population health is to address a broader range of factors that impact the health of different populations. This improves the health outcomes of individuals, including the distribution of such outcomes within the group. (Kindig & Stoddart, 2003)

Slide 5: Population health is…

- Population health is an interdisciplinary collaboration to improve the health status of a defined population.
  - Bring scientists, clinicians, payers, & public together to address health needs of the population.
  - Focus on use of analytics to provide evidence for creating personalized health recommendations.
  - Focus on addressing health equity and social determinants of health through community led priorities
  - Focus on developing interventions to improve the health of a population

Slide 6: Population health sciences

- The Institute of Health Care Improvement describes population health sciences as the design, delivery, coordination and payment of high-quality health care services to manage the triple aim for a population using the best resources we have available to us within the health care system. (Institute of Health Care Improvement)
- The Triple Aim in this case is 1) improving patients experiences 2) improving the health of population and 3) reducing per capital costs of health care

Slide 7: Examples of the importance of population health

- The next 5 slides provide examples of the importance of population health using the lenses of social determinants of health and the burden of chronic diseases
  - Session organizers might consider providing these slides to participants prior to the session or at the beginning of the session to orient participants to the topic.
  - Session organizers might consider removing these slides if participants are already familiar with population health.

Slide 8: Why is population health important? (Schroeder, 2007)

- Here we discuss how health, living and death is affected by various factors including genetics, social circumstances, environmental exposure, healthcare and behavioural patterns. You will see with this graph on the left that genetics and health care only make up 40% of what affects premature death in the US. In fact, behavioural causes alone account for 40% of all deaths in the US.
- The graph on the right represents various behavioural causes that affect premature death in the US; you can see here that obesity/ inactivity and smoking make up a significant portion of these behaviors.
- This helps us visualize why population health is important and what areas we can look towards first.

Slide 9: Social Determinants of Health (World Health Organization)

Social determinants of health are the non-medical factors that influence health outcomes. They are the conditions in which people are born, grow, work, live, and age, and the wider set of forces and systems shaping the conditions of daily life. These forces and systems include economic policies and systems, development agendas, social norms, social policies and political systems.

- Race
- Ethnicity
- Sexuality
- Gender Identity
- Gender
- Environment (where live and/or work)
- Poverty / Resources
- Literacy / Numeracy
- Early Life
- Stress
- Social gradient (social ladder)

Slide 10: How can health be affected by social determinants of health? (World Health Organization, 2021)

- Individuals with more resources and better social circumstances, such as increased income or education or literacy or those less early life stress tend to have the most power and resources, and on average live longer and healthier lives. Those without have the least power and usually run at least twice the risk of serious illness and premature death in comparison ~~top~~_~~.~~_
- Social determinants of health affect all aspects of health as seen below
- Life expectancy
- Infant mortality
- Death rates
- Morbidity
- Disability
- Quality of life
- Self-assessed health
- Happiness and well-being

Slide 11: Burden of Chronic Disease in the US (National Center for Chronic Disease Prevention

and Health Promotion, 2021)

- Chronic diseases are the leading causes of morbidity and mortality in the US. Additionally, they cost trillions to the health care system (3.8 trillion).
- These diseases include heart disease, cancer, chronic lung disease, stroke, Alzheimer’s Disease, diabetes, and chronic kidney disease.
- 6 in 10 adults in the US have one of these diseases, 4 in 10 have two or more chronic disease
- Chronic disease as a whole, or each of these diseases individually, are just some examples of how population health can drastically affect the health of a population and community

Slide 12: Lifestyle risks include: (National Center for Chronic Disease Prevention

and Health Promotion, 2021)

- Tobacco use, poor nutrition, lack of physical activity and excessive alcohol use are just four of the lifestyle factors that heavily influence chronic disease

Slide 13: Preparation for the case activity

- The next 11 slides provide content that introduces the role of the academic health centers in population health and prepares participants for the case activity.
- After the case stem, you will find slides that detail the ignite session prompts with an additional slide of information to help brainstorm ideas for each ignite session
- Session organizers may prefer to go through all these slides as a big group or to break into small groups for the remainder of this information

Slide 14: Population health at an Academic Health Center (AHC) (Gourevitch et al., 2019)

- AHCs can play a central role in supporting population health. For example, it can support clinical care in practice redesign. This could be creating programs for patient centred medical care, coordination of patients with complex needs and chronic illnesses, coordination between speciality and primary care. It can help with patient engagement and can be used to drive analytics and ensure a trained workforce.

Slide 15: Successful implementation of population health initiatives include:

AHCs to succeed or leverage its capacity as population health partner, these four elements are critical

- As this activity unfolds, we will see that population health sciences can support and be supported by an AHC through various domains; today we will discuss analytics, community engagement, education and implementation.

Slide 16: Case stem

More detailed instructions on conducting the below case activity are provided in the accompanying educational summary report.

Congratulations on your recent appointment as Chief Executive Officer (CEO) of University Health System (UHS), a growing academic health system in Anytown. UHS comprises 4 hospitals (1 tertiary/teaching, 3 community), 4 regional specialty centers, and 10 primary care practices located in the tri-county area. Care is delivered in UHS by over 1500 faculty physicians and advanced practice providers. In addition, like many AHCs, hundreds of residents, fellows, and students learning in the health professions deliver large portions of care at UHS. UHS owns University Health Plans, which operates both a Managed Medicaid Plan and a Medicare Advantage Plan.

Your first six months as CEO of UHS proceed well, as you learn about your new organization, its people and programs, the community, and external stakeholders. Yesterday, the Mayor of Anytown shares with you that at the recent National Meeting of Mayors, presenters reviewed health outcomes data, and Anytown was unfortunately highlighted as having lower quality and higher cost health care than other similar cities in the country. The Mayor asks you as the new CEO of one of the nation’s leading AHCs to assist her, in partnership with private and government Payers and Anytown governmental agencies, in better assessing the health of the citizens that live in and near Anytown, and work with her and her administration to implement strategies that substantially improve the population’s health.

Slide 17 –Small Group Prompt – Analytics

The Mayor pledges the support of government analysts and data sets, and as CEO of UHS, you offer the assistance of your analytics team as well. Working with your colleagues in this breakout session, identify two opportunities afforded by the structure of an academic health center in taking an analytics approach to population health initiatives, and two barriers?

Slide 18 – Analytics and Population Health

- Application of quantitative methods to analyze current and historical data to gain insights about a group or to determine strategies for approaching health issues.
- AHCs can utilize analytics to:
  - Assess a group’s health status and evaluate the implementation of population health initiatives.
  - Predict health trends
  - Improve and target outreach
  - Optimize health outcomes of groups
- For example:
  - Using the information from patient reported outcomes (PROs) to change physician practices and improve patient care (e.g., patient expectations).

Slide 19 – Small Group Prompt – Community Engagement

The Mayor asks you and UHS to engage with the Anytown community in a population health improvement initiative. Working with your colleagues in this breakout session, identify two opportunities afforded by the structure of an academic health center in engaging communities in population health initiatives, and two barriers?

Slide 20 – Community Engagement and Population Health

- Community engagement is the active partnership of the local community with the AHC in the planning, design, and implementation of health care services, research, and training.
- AHCs can partner with their community to
  - Tailor its services to the local community’s needs and values
  - Improve health outcomes
  - Increase the likelihood that offered services will be accepted by the community
- For example
  - When considering a new blood pressure screening initiative, AHC staff actively partner with community members to understand community needs and values and to ultimately implement the initiative.

Slide 21 –Small Group Prompt – Education and Training

As the major education and training provider of health professionals for Anytown and the surrounding regions, the Mayor asks how you can prepare your graduates to take better care of the population living in Anytown. Working with your colleagues in this breakout session, identify two opportunities afforded by the structure of an academic health center to advance population health training in your education programs, and two barriers?

Slide 22 – Education and Training and Population Health

- Education and training are critical across the continuum of health professions education (e.g., for nursing students, doctoral students, practicing physicians)
  - Can include coverage of population health principles and training to understand, apply, and conduct population health research
- For example:
  - An AHC launches a population health pathway for medical students integrated across their curriculum with an emphasis on the role of population health in their roles as future physicians.

Slide 23 –Small Group Prompt – Implementation

Initial analysis by the Anytown health department identifies diabetes as a significant health problem. UHS has a strong diabetes care program. Working with your colleagues in this breakout session, identify two opportunities afforded by the structure of an academic health center to implement your diabetes program throughout Anytown, and two barriers?

Slide 24: Implementation and Population Health

- Implementation is the process of putting an initiative into effect in a health care system or in the community
  - Implementation requires that AHC work collaboratively with community members to purposefully, using a series of predetermined steps, put into practice activities and/or programs departments

- For example:
  - With the aim of improving oral cancer screening, the AHC coordinates with the local health officials to launch a mobile health van program that includes dental residents.

References:

Gourevitch, M. N., Curtis, L. H., Durkin, M. S., Fagerlin, A., Gelijns, A. C., Platt, R., . . . Tierney, W. M. (2019). The Emergence of Population Health in US Academic Medicine: A Qualitative Assessment. *JAMA Netw Open, 2*(4), e192200. doi:10.1001/jamanetworkopen.2019.2200

Kindig, D., & Stoddart, G. (2003). What is population health? *Am J Public Health, 93*(3), 380-383. doi:10.2105/ajph.93.3.380

Schroeder, S. A. (2007). Shattuck Lecture. We can do better--improving the health of the American people. *The New England journal of medicine, 357*(12), 1221-1228. doi:10.1056/NEJMsa073350

Centers for Disease Control, Social Determinants of Health https://www.cdc.gov/socialdeterminants/index.htm

Institute of Health Care Improvement

http://www.ihi.org/Engage/Initiatives/TripleAim/Pages/default.aspx

National Center for Chronic Disease Prevention and Health Promotion

<https://www.cdc.gov/chronicdisease/resources/infographic/chronic-diseases.htm>

National Center for Chronic Disease Prevention and Health Promotion: https://www.cdc.gov/chronicdisease/about/costs/index.htm

World Health Organization, About Social Determinants of Health

<http://www.who.int/social_determinants/sdh_definition/en/>.
