# Supplementary material for: Population Health at the Academic Health Center: An Interactive, Multipart, Case-Based Session for Executives, Faculty, and Administrators
Source: MedEdPORTAL. 2022 Jan 7;18:11204. doi: 10.15766/mep_2374-8265.11204 (PMC8738160; doi:10.15766/mep_2374-8265.11204)
Supplement: Supplementary file 1 — Call for Abstracts.docxReviewer Rubric.docxCase Stem and Small-Group Prompts.docxSession Evaluation.docxIntroduction to Population Health.pptxFacilitator Guide.docx [file mep_2374-8265.11204-s001.zip › C. Case Stem and Small-Group Prompts.docx]

**Population Health at Academic Health Centers**

The case and case stems distributed to participants in the Population Health at the Academic Health Center: An interactive multi-part, case-based session for executives, faculty and administrators. Individuals utilizing this case at a single academic health center might consider tailoring some of the case details to their local context.

Case

Congratulations on your recent appointment as Chief Executive Officer (CEO) of University Health System (UHS), a growing academic health system in Anytown. UHS comprises 4 hospitals (1 tertiary/teaching, 3 community), 4 regional specialty centers, and 10 primary care practices located in the tri-county area. Care is delivered in UHS by over 1500 faculty physicians and advanced practice providers. In addition, like many AHCs, hundreds of residents, fellows, and students learning in the health professions deliver large portions of care at UHS. UHS owns University Health Plans, which operates both a Managed Medicaid Plan and a Medicare Advantage Plan.

Your first six months as CEO of UHS proceed well, as you learn about your new organization, its people and programs, the community, and external stakeholders. Yesterday, the Mayor of Anytown shares with you that at the recent National Meeting of Mayors, presenters reviewed health outcomes data, and Anytown was unfortunately highlighted as having lower quality and higher cost health care than other similar cities in the country. The Mayor asks you as the new CEO of one of the nation’s leading AHCs to assist her, in partnership with private and government Payers and Anytown governmental agencies, in better assessing the health of the citizens that live in and near Anytown, and work with her and her administration to implement strategies that substantially improve the population’s health.

Case stem distributed to participants in the analytics group

The Mayor pledges the support of government analysts and data sets, and as CEO of UHS, you offer the assistance of your analytics team as well. Working with your colleagues in this breakout session, identify two opportunities afforded by the structure of an academic health center in taking an analytics approach to population health initiatives, and two barriers?

Case stem distributed to participants in the communication group

The Mayor asks you and UHS to engage with the Anytown community in a population health improvement initiative. Working with your colleagues in this breakout session, identify two opportunities afforded by the structure of an academic health center in engaging communities in population health initiatives, and two barriers?

Case stem distributed to participants in the education and training group

As the major education and training provider of health professionals for Anytown and the surrounding regions, the Mayor asks how you can prepare your graduates to take better care of the population living in Anytown. Working with your colleagues in this breakout session, identify two opportunities afforded by the structure of an academic health center to advance population health training in your education programs, and two barriers?

Case stem distributed to participants in the implementation group

Initial analysis by the Anytown health department identifies diabetes as a significant health problem. UHS has a strong diabetes care program. Working with your colleagues in this breakout session, identify two opportunities afforded by the structure of an academic health center to implement your diabetes program throughout Anytown, and two barriers?
