# Supplementary material for: Population Health at the Academic Health Center: An Interactive, Multipart, Case-Based Session for Executives, Faculty, and Administrators
Source: MedEdPORTAL. 2022 Jan 7;18:11204. doi: 10.15766/mep_2374-8265.11204 (PMC8738160; doi:10.15766/mep_2374-8265.11204)
Supplement: Supplementary file 1 — Call for Abstracts.docxReviewer Rubric.docxCase Stem and Small-Group Prompts.docxSession Evaluation.docxIntroduction to Population Health.pptxFacilitator Guide.docx [file mep_2374-8265.11204-s001.zip › Educational_Summary_Report_11204.pdf]

MedEdPORTAL® The AAMC Journal of Teaching and Learning Resources

Original Publication Open Access

Population Health at the Academic Health Center: An Interactive, Multipart, Case-Based Session for Executives, Faculty, and Administrators

Lauren A. Maggio, PhD*, Federica S. Brecha, Angela Fagerlin, PhD, Michael L. Good, MD, Steven L. Kanter, MD

*Corresponding author: lauren.maggio@usuhs.edu

Abstract

Introduction: Academic health centers (AHCs) play critical roles in population health by providing health care, conducting population health research, and providing population health training and education. This publication describes an interactive, multipart, case-based session targeted at AHC executives, faculty, and administrators about population health initiatives at AHCs and how the AHC structure can be leveraged to promote population health. **Methods:** This 90-minute virtual session was conducted during the Association of Academic Health Centers' 2020 annual meeting. The session opened with 5-minute, Ignite-style presentations showcasing population health innovations. Next, in small groups, participants discussed a case introducing a fictional AHC charged with assisting its local government's population health efforts. Participants self-selected into one of four small groups (analytics, education and training, community engagement, and implementation) and were provided additional case content and tailored prompts. In the large group, participants debriefed their discussions. Participants completed a postsession survey. **Results:** Forty-six individuals from 31 AHCs participated. Eighteen participants completed the survey. Sixteen respondents (89%) agreed the session was valuable and provided ideas for implementation at their AHC. Fifteen participants (83%) indicated that they planned to follow up with their colleagues regarding how to leverage the AHC structure to improve population health. **Discussion:** This session provides an interactive forum to discuss population health in the context of an AHC and examine how its structure can facilitate population health. While offered at a conference, the session can also be implemented at a single AHC to foster local understanding of population health and inform future initiatives.

Keywords
Population Health, Community Engagement, Case-Based Learning, Community-Based Medicine

Educational Objectives

By the end of this activity, learners will be able to:

1. Discuss population health in the context of the academic health center (AHC).
2. Identify population health approaches to adopt for their own AHC.
3. Describe how the structure of an AHC can impact its role in promoting population health.

Introduction

According to the United States Centers for Disease Control website, "We all have a stake in population health."[1] In concurrence with population health advocates,[2,3] we propose that academic health centers (AHCs) are key stakeholders in advancing population health. To this end, AHCs play multifaceted and interconnected roles, including those related to providing health care, conducting population health research, and offering training and education in population health. Thus, in this publication, we describe an interactive session for AHC executives, faculty, and administrators to learn about population health initiatives at AHCs and discuss how the structure of the AHC can be leveraged to promote population health.

AHCs are defined as "academic institutions that include a medical school, one or more additional health professions schools or programs, and an owned or affiliated hospital/health system."[4] Often serving as anchor institutions, AHCs make considerable commitments of financial, human, and intellectual resources to address social challenges in their local communities.[5] Related to this, AHCs have expanded population health initiatives, including those related to education and training. In recent studies, authors have described the rise of department-level population health

Citation:
Maggio LA, Brecha FS, Fagerlin A, Good ML, Kanter SL. Population health at the academic health center: an interactive, multipart, case-based session for executives, faculty, and administrators. *MedEdPORTAL*. 2022;18:11204.
https://doi.org/10.15766/mep_2374-8265.11204

Copyright © 2022 Maggio et al. This is an open-access publication distributed under the terms of the Creative Commons Attribution license.

initiatives at AHCs in the US[6] and the presence of population health in most medical school curricula.[7] In addition to these initiatives, medical educators have published educational materials in *MedEdPORTAL* addressing population health topics. For example, there are several educational resources available that focus on population health through the lens of a specific event or condition, including pandemics,[8] HIV,[9] and community nutrition.[10]

While valuable, the varied educational initiatives undertaken by AHCs have been primarily targeted towards undergraduate health professions students or those in graduate training. We propose that to optimize population health, initiatives should permeate the entire enterprise and that training will be beneficial for all AHC stakeholders. Currently, we are unaware of training that targets AHC leadership, including executives, faculty, and administrators. This gap suggests an opportunity for the training described herein that details the implementation of a multicomponent, case-based, interactive session for executives, faculty members, and administrators at AHCs. In this training, we provide participants the opportunity to learn about ongoing population initiatives at AHCs and engage them in discussion about the unique opportunities afforded by AHC structures to promote population health.

Methods

We conducted a 90-minute session that included Ignite-style presentations[11] on population health innovations and an interactive, case-based, small-group breakout activity. The session was a component of the Association of Academic Health Centers (AAHC) 2020 annual meeting. Due to the 2020 coronavirus pandemic, the AAHC annual meeting was conducted virtually via a videoconferencing platform (Zoom).

The session was envisioned and developed by members of the AAHC President's Council on Health Insurance and Population Health as a component of their charge to introduce the AAHC members to innovations and best practices in population health. Over 9 months, the 12-member council, composed of AHC executives, met via conference call to design the structure of the session and draft the educational materials. Dr. Michael L. Good, the Chief Executive Officer (CEO) at University of Utah Health, chaired the President's Council.

Participants

The target audience included leaders (e.g., CEOs, chancellors, presidents, provosts), faculty members, and administrators from all AAHC member institutions, a group of more than 125 AHCs located in 29 countries. Participants were not required to have any prerequisite knowledge. The session was advertised as a component of the annual meeting through the association's newsletter and meeting registration site. There was no fee to attend the meeting or session. While this particular offering of the educational session was implemented at a professional meeting, the session and its related support materials can be presented in multiple contexts, including at a single AHC, where the session could be offered center-wide or as an initiative across multiple departments.

Each of the four small groups was led by two facilitators: a member of the AAHC President's Council and a member of the Population Health Leaders in Academic Medicine (PHLAM). PHLAM is an organization composed of roughly 35 AHC department chairs and deans of population health. All facilitators were based in the US. We recruited the eight facilitators prior to the session via an email invitation to the two groups.

Implementation

The 90-minute session was divided into two main components: Ignite-style presentations[11] and an interactive, case-based activity. To begin, the President's Council chair, Dr. Michael L. Good, acted as the large-group facilitator and presented the following session outline:

- Welcome and overview of session agenda and objectives (5 minutes).
- Ignite-style presentations (35 minutes).
- Brief transition to small-group session (5 minutes).
- Small-group breakout session (20 minutes).
- Large-group discussion (20 minutes).
- Concluding remarks (5 minutes).

Ignite-style presentations: Seven AHC faculty and executives each presented an Ignite-style presentation.[11] Ignite presentations are rapid-paced presentations conducted in 5 minutes or less in which participants are allowed a limited amount of PowerPoint slides that are advanced automatically, usually in 20-second intervals. In this instance, presenters were limited to five PowerPoint slides, together accounting for 35 minutes of the overall session. Unlike a traditional Ignite session, participants were permitted to use the allotted time as they liked without a timed sequential forwarding of slides. These short presentations provided session participants with brief glimpses into population health initiatives that had been implemented at the presenters' AHCs. This portion of the session was aimed at helping participants identify population health approaches that could be adopted at their own AHC. Presentations ranged from descriptions of advances in measuring disparities across a state

Copyright © 2022 Maggio et al. This is an open-access publication distributed under the terms of the Creative Commons Attribution license.

to the development of intensive outpatient clinics. Abstracts for all seven presented initiatives are publicly available on the AAHC website.[12]

Two months prior to the session, the AAHC President's Council on Health Plans and Population Health circulated a call for abstracts to its membership to present population health initiatives from their AHCs. Abstracts were limited to 300 words, including a description of the problem addressed by the initiative, a description of how the initiative was implemented, and any achieved or intended outcomes. Additionally, in the spirit of learning from each other, submitters were encouraged not only to report initial success but also to describe elements of their projects that surprised and challenged the AHC and how the challenges were addressed (Appendix A includes the call for abstracts). Using an anonymized review process, President's Council members selected the seven presentations out of 21 submitted abstracts. Abstracts were judged based on (1) the potential level of broad interest to AAHC members, (2) the clarity of the abstract, (3) the relevance of the initiative's outcomes to improving population health at an AHC, and (4) whether the lessons learned from the initiative and how they were addressed could help AAHC members advance their thinking on population health. The review process was operationalized using Survey Monkey, and each submitted abstract was scored by at least two reviewers (Appendix B includes the reviewer form).

Case-based activity: During the second half of the session, participants engaged in a case-based activity that lasted 45 minutes and included both small- and large-group interactions. To start this component, participants were automatically transferred from the main Zoom room into one of four breakout sessions using Zoom's built-in functionality. Upon registering for the session, participants selected their breakout rooms, which were set up to focus on key elements of population health, specifically, analytics, community engagement, education and training, and implementation. Participants did not receive any additional information about the topics and were asked to select based on their interests.

The fictional case (Appendix C) was written by Michael L. Good and Lauren A. Maggio with input from President's Council and PHLAM members. The case was presented in two phases. First, all participants received a main case stem that was approximately a half-page of text. The case stem set up the situation by describing a request to an AHC CEO from the mayor of the city in which their AHC was located. The CEO was asked to assist in assessing the health of the citizens and to partner with the mayor and her administration to implement strategies to substantially improve the population's health. This case stem was distributed by email to all session participants 24 hours prior to the session to enable them to familiarize themselves with the content. Facilitators received the final case 1 week prior to the session.

The second phase of the case was tailored to the four small-group themes and included additional brief case details and prompts to facilitate discussion (Appendix C). Participants received the second phase of the case once they had assembled in their small-group virtual breakout rooms. The case stem plus the second phase of the case was projected as a slide to all participants. Once in their small groups, participants were instructed to read the additional case content and to discuss the case in relation to the provided prompts and their group's theme. The prompts asked participants to identify two opportunities and two barriers afforded by the structure of an AHC in light of their group's theme to undertaking population health initiatives. During the discussion, the facilitators were asked to identify a volunteer to report back to the large group based on the prompts. During the small-group activity, the two facilitators participated in the discussion and helped participants stay on track regarding their task. Facilitators were not expected to provide content or act as experts in the small-group session but were available for answering questions about a group's theme and the case.

After 20 minutes, the participants were all automatically returned to the large-group Zoom session. Each group briefly reported on its discussion of the group's prompts. After reporting, groups were encouraged to discuss synergies and overlaps between the themes and to brainstorm potential opportunities. The large-group facilitator closed the session and thanked the participants.

Evaluation

The educational activity was evaluated using a 10-question, anonymous survey (Appendix D) that was designed to collect participants' reactions to the activity and their future intentions of integrating the learned content into their work roles. The survey was emailed to the participants immediately following the session. Participants were asked to self-report on how useful they found the session and their intentions to apply new knowledge at their AHC. The survey was designed by members of the President's Council and AAHC staff with expertise in survey design. The survey was operationalized using Survey Monkey. Participants were sent a total of two requests to complete the survey. The University of Utah Health Institutional Review Board reviewed this initiative and deemed it non-human subjects research.

Copyright © 2022 Maggio et al. This is an open-access publication distributed under the terms of the Creative Commons Attribution license.

Future Implementation
The above methods describe the session as presented at a professional conference. To facilitate implementation of the session at a single AHC, which may be open to a variety of stakeholders, we have crafted an additional background PowerPoint presentation (Appendix E) and accompanying facilitator guide (Appendix F) providing logistical details for implementation and introducing major concepts in population health to be integrated as necessary.

Results

On October 15, 2020, 38 learners and eight facilitators participated in the educational activity. Overall, the 46 participants represented 31 AHCs located in seven countries. The majority of participants were located in the US ($n = 37$). There were also two participants from the Association of American Colleges.

Learners included AHC CEOs, chancellors, deans, department chairs, vice provosts, and faculty members. Learners self-selected into the following small groups: analytics ($n = 8$), community engagement ($n = 12$), education and training ($n = 7$), and implementation ($n = 11$).

Eighteen participants completed the survey for a 39% response rate. Results are summarized in the Table. Overall, 89% of participants ($n = 16$) agreed that the session was valuable for their roles at their AHC. In terms of future action, the majority of participants reported that colleagues' shared presentations provided ideas for implementation at their own institutions ($n = 16$; 89%) and that they planned to follow up with members of their own community after the session ($n = 15$; 83%). Specific to the small-group breakout session, 83% ($n = 15$) indicated that these targeted sessions provided with them ideas for how to leverage the structure of an AHC to improve population health.

At the end of the survey, participants were provided with a comments box to describe which aspects of the session they found most or least effective. Fourteen participants commented. Overall, the comments were positive. For example, one faculty member commented: "I thought the format was great! I appreciated hearing from a wide range of [AHCs] and the structure of the breakout rooms with the moderators were excellent." A CEO noted that "learning about approach and strategies used at peer AHC's" was effective. Participants also provided constructive feedback, with one chancellor advising, "It would be nice if, in [the] next session, you would give [a] case in a situation where [the] academic health system is in a developing country." Lastly, several participants noted that they wished that the session had been longer, with additional time allotted to the small groups and for posing questions to Ignite-style speakers.

Discussion

We developed an interactive, multipart session on population health that was well received by participants and met the proposed learning objectives. This session format can be replicated and used to facilitate executives, faculty, and administrators in discussing and understanding how population health initiatives have been implemented and the ways in which the structure of an AHC can impact population health. Additionally, the session provided participants with actionable ideas to consider for implementation at their own AHC and appears to have inspired future action.

Reflection
Our session was developed during the 2020 coronavirus pandemic, which influenced its design and implementation.

Table. Summary of Participant Responses to a Postsession Survey ($N = 18$)[a]

Statements	No. (%)			
	Strongly Agree	Agree	Neutral	Left Blank
This population health session was valuable to my role at my academic health center.	12 (67)	4 (22)	2 (11)	
I will apply knowledge gained from this population health session at my academic health center.	10 (56)	6 (33)	2 (11)	
Hearing colleagues describe population health initiatives at their academic health center provided me ideas to implement at my own academic health center.	13 (72)	3 (17)	1 (6)	1 (6)
I will follow up with members of my academic health center regarding information shared in this population health session.	12 (67)	3 (17)	2 (11)	1 (6)
Before the population health breakout session, I appreciated how the structure of an academic health center could impact its role in promoting population health.	8 (44)	8 (44)	2 (11)	
After the population health breakout session, I appreciated how the structure of an academic health center could impact its role in promoting population health.	13 (72)	4 (22)	1 (6)	
The breakout session provided me ideas for how to leverage the structure of my academic health center to impact population health	12 (67)	3 (17)	3 (17)	

[a]Participants were offered the options of *strongly agree*, *agree*, *neutral*, *disagree*, and *strongly disagree*. However, no participant selected *disagree* or *strongly disagree* for any of the statements.

Copyright © 2022 Maggio et al. This is an open-access publication distributed under the terms of the Creative Commons Attribution license.

Throughout the planning, we considered the features and limitations of our virtual platform, Zoom. For example, we leveraged the breakout room feature, which enabled us to automatically assign participants to smaller groups to facilitate targeted conversations. On the other hand, this reliance on technology, which can malfunction at times, necessitated additional planning. To head off technology issues, we asked all speakers to sign in to Zoom 25 minutes early to a virtual speaker ready room. While this added a burden for our speakers, we felt that it facilitated a smooth session overall and ensured that speakers were informed and at ease during the session. We also provided all speakers with a detailed run-of-show document with the steps described in our implementation section to ensure that they were apprised of the overall flow of the event.

In addition to potential glitches, we were sensitive that during the pandemic most of our participants were logging many hours on video conferences, which could lead to Zoom fatigue.[13] With this in mind, we planned the session to be only 90 minutes. Additionally, we attempted to keep the session moving at all times and provided varied formats (e.g., large- and small-group activities, brief Ignite-style presentations). In future iterations, educators can consider extending any of the session components. For example, we received feedback that the participants would have liked additional time in their small groups. Additionally, while follow-up questions were asked and answered using the Zoom chat feature simultaneously during subsequent presentations, participants suggested that a brief, live (non-chat, nonsimultaneous) question-and-answer component following each Ignite-style presentation would have been valuable. Lastly, while this was conducted as an online session, upon reflection we felt that a key component of the session was the human interactions, including our opportunity to integrate PHLAM members into our small-group discussions. We encourage those considering offering such a session to invite population health faculty and researchers from their own AHC in addition to external stakeholders to provide additional insights to the session.

While we recognize that the evaluation data presented in this article provide evidence for conference implementation, we believe that the participants' reported satisfaction with the session and their plans to implement material from the session remain applicable to alternative implementations, such as at a single AHC. To facilitate implementation of the session at a single AHC, which may be open to a variety of stakeholders, we have crafted an additional background PowerPoint presentation (Appendix E) and accompanying facilitator guide (Appendix F) providing logistical details for implementation and introducing major concepts in population health to be integrated as necessary. Additionally, where appropriate in the supplemental appendices, we have provided brief annotations that describe how the materials could be adapted for use at a single AHC. For example, the Ignite sessions could be used for highlighting population health innovations from across the AHC to promote awareness of local initiatives and potentially foster interinstitutional collaboration. The case study could also be discussed in relation to a single AHC, with the opportunity to tailor the case to the local context. This tailoring could potentially increase the case's relevance and spark a cross-institutional conversation about population health and future directions at the AHC.

Limitations

While the session was well received by those attending, there were limitations to our initiative. One limitation was that the case used for the small-group component was drafted within the context of an AHC in the US (e.g., the case stem mentioned Medicare). As noted in one participant's feedback, this design choice could have potentially lessened the value of the session for participants based outside the US. However, we propose that the materials could be readily adapted to the culture in which the session is presented or could be made generic. For those contemplating adopting this session in the future, they might consider partnering with international stakeholders to ensure that the content is relevant to their anticipated population.

There were also limitations to our evaluation efforts. Our survey provided only the participants' reactions to the session (i.e., Kirkpatrick level one impact data[14]). Our survey had a response rate of 39%, which, although low, aligns with research reporting that health professional survey response rates are commonly less than 20%.[15] A more robust approach for future researchers may be to follow up with participants, potentially over a longitudinal time frame, to understand whether and how participants were able to implement their understanding and ideas within the context of their AHC. This could be achieved through follow-up interviews or an additional survey, which could also seek to measure higher-level outcomes, such as skill and behavior changes.

Future Directions

Based on the positive reception of the session and participants' intentions to act upon what was learned, we feel that there is evidence to warrant reimplementing the session in other settings and continuing to build upon the model. While future educators can consider offering this session as currently designed, we

Copyright © 2022 Maggio et al. This is an open-access publication distributed under the terms of the Creative Commons Attribution license.

encourage them to optimize it for their unique context, including offering the session at their specific AHC, as noted above. To this end, in creating this session we aimed to ensure that our session was replicable and flexible. For example, the two main components of the session can be decoupled if necessary due to time constraints or a desire to focus on a specific component. Additionally, as designed, this session can be offered in a virtual or in-person format. Looking to the future, while this session was targeted towards the AHC community, we believe there is also opportunity to include additional stakeholders in the conversation (e.g., community participants, local government officials, health professions students) to enrich discussions.

Appendices

A. Call for Abstracts.docx

B. Reviewer Rubric.docx

C. Case Stem and Small-Group Prompts.docx

D. Session Evaluation.docx

E. Introduction to Population Health.pptx

F. Facilitator Guide.docx

All appendices are peer reviewed as integral parts of the Original Publication.

Lauren A. Maggio, PhD: Programs Scholar, Association of Academic Health Centers; Professor, Department of Medicine, Uniformed Services University; ORCID: https://orcid.org/0000-0002-2997-6133

Federica S. Brecha: Fourth-Year Medical Student, University of Utah School of Medicine

Angela Fagerlin, PhD: Professor and Chair, Department of Population Health Sciences, University of Utah; Research Scientist, Salt Lake City Veterans Affairs Informatics Decision-Enhancement and Analytic Sciences (IDEAS) Center for Innovation

Michael L. Good, MD: Chief Executive Officer, University of Utah Health; Dean, University of Utah School of Medicine; A. Lorris Betz Senior Vice President, Health Sciences, University of Utah

Steven L. Kanter, MD: President and Chief Executive Officer, Association of Academic Health Centers

Acknowledgments
We would like to thank the facilitators of the small-group sessions from the Population Health Leaders in Academic Medicine group, Dr. Tracie Collins, Dr. Edward Ellerbeck, and Dr. William Tierney; and from the President's Council, Dr. David Lubarsky, Dr. Janis Orlowski, Dr. Cameron Patterson, and Dr. Mark Stacey.

Disclosures
None to report.

Funding/Support
None to report.

Prior Presentations
Good ML, Maggio LA. AAHC President's Council on Population Health. Workshop presented virtually at: Association of Academic Health Centers Annual Meeting; October 16, 2020.

Ethical Approval
The University of Utah Health Institutional Review Board deemed further review of this project not necessary.

Disclaimer
The views expressed in this presentation are those of the authors and do not necessarily reflect the official policy or position of the Uniformed Services University of the Health Sciences, the Department of Defense, or the US Government.

References

1. What is population health? Centers for Disease Control and Prevention. Updated October 6, 2020. Accessed January 29, 2021. https://www.cdc.gov/pophealthtraining/whatis.html
2. Gourevitch MN. Population health and the academic medical center: the time is right. *Acad Med*. 2014;89(4):544-549. https://doi.org/10.1097/ACM.0000000000000171
3. Smitherman HC Jr, Baker RS, Wilson MR. Socially accountable academic health centers: pursuing a quadripartite mission. *Acad Med*. 2019;94(2):176-181. https://doi.org/10.1097/ACM.0000000000002486
4. Wartman SA, Zhou Y, Knettel AJ. Health reform and academic health centers: commentary on an evolving paradigm. *Acad Med*. 2015;90(12):1587-1590. https://doi.org/10.1097/ACM.0000000000000948
5. Koh HK, Bantham A, Geller AC, et al. Anchor institutions: best practices to address social needs and social determinants of health. *Am J Public Health*. 2020;110(3):309-316. https://doi.org/10.2105/AJPH.2019.305472
6. Gourevitch MN, Curtis LH, Durkin MS, et al. The emergence of population health in US academic medicine: a qualitative assessment. *JAMA Netw Open*. 2019;2(4):e192200. https://doi.org/10.1001/jamanetworkopen.2019.2200
7. Morse R, Smith A, Fitzgerald-Wolff S, Stoltzfus K. Population health in the medical school curriculum: a look across the country. *Med Sci Educ*. 2020;30(4):1487-1493. https://doi.org/10.1007/s40670-020-01083-z
8. Jackson JM, Shen E, Peters TR. The zombie virus pandemic: an innovative simulation integrating virology, population health, and bioethics for preclinical medical students. *MedEdPORTAL*. 2020;16:11016. https://doi.org/10.15766/mep_2374-8265.11016

Copyright © 2022 Maggio et al. This is an open-access publication distributed under the terms of the Creative Commons Attribution license.

9. Sell J, George D, Levine MP. HIV: a socioecological case study. *MedEdPORTAL*. 2016;12:10509. https://doi.org/10.15766/mep_2374-8265.10509
10. Lang RD, Jennings MC, Lam C, Yeh HC, Zhu C, Kumra T. Community culinary workshops as a nutrition curriculum in a preventive medicine residency program. *MedEdPORTAL*. 2019; 15:10859. https://doi.org/10.15766/mep_2374-8265.10859
11. Ignite. Ignite Talks. Accessed September 29, 2021. http://www.ignitetalks.io/
12. AAHC President's Council on Health Plans and Population Health. Association of Academic Health Centers. Accessed January 29, 2021. https://www.aahcdc.org/Initiatives/Presidents-Councils/AAHC-Presidents-Council-on-Health-Plans-and-Population
13. Fosslien L, Duffy MW. How to combat Zoom fatigue. *Harvard Business Review*. April 29, 2020. https://hbr.org/2020/04/how-to-combat-zoom-fatigue
14. Kirkpatrick DL. *Evaluating Training Programs: The Four Levels.* 2nd ed. Berrett-Koehler Publishers; 1998.
15. Dykema J, Jones NR, Piché T, Stevenson J. Surveying clinicians by web: current issues in design and administration. *Eval Health Prof*. 2013;36(3):352-381. https://doi.org/10.1177/0163278713496630

Received: April 26, 2021
Accepted: September 10, 2021
Published: January 7, 2022

Copyright © 2022 Maggio et al. This is an open-access publication distributed under the terms of the Creative Commons Attribution license.
